# Association of hypoglycaemia with the risks of arrhythmia and mortality in individuals with diabetes - a systematic review and meta-analysis

**DOI:** 10.3389/fendo.2023.1222409

**Published:** 2023-08-14

**Authors:** Gangfeng Li, Shuping Zhong, Xingmu Wang, Fuyuan Zhuge

**Affiliations:** ^1^ Clinical Laboratory Center, Shaoxing People’s Hospital, Shaoxing, Zhejiang, China; ^2^ Department of Hospital Management, Shaoxing People’s Hospital, Shaoxing, Zhejiang, China; ^3^ Department of Endocrine and Metabolism, Shaoxing People’s Hospital, Shaoxing, Zhejiang, China

**Keywords:** hypoglycaemia, diabetes, arrhythmia, mortality, risk, systematic review, meta-analysis

## Abstract

**Background:**

Hypoglycaemia has been linked to an increased risk of cardiac arrhythmias by causing autonomic and metabolic alterations, which may be associated with detrimental outcomes in individuals with diabetes(IWD), such as cardiovascular diseases (CVDs) and mortality, especially in multimorbid or frail people. However, such relationships in this population have not been thoroughly investigated. For this reason, we conducted a systematic review and meta-analysis.

**Methods:**

Relevant papers published on PubMed, Embase, Cochrane, Web of Knowledge, Scopus, and CINHAL complete from inception to December 22, 2022 were routinely searched without regard for language. All of the selected articles included odds ratio, hazard ratio, or relative risk statistics, as well as data for estimating the connection of hypoglycaemia with cardiac arrhythmia, CVD-induced death, or total death in IWD. Regardless of the heterogeneity assessed by the I^2^ statistic, pooled relative risks (RRs) and 95% confidence intervals (CI) were obtained using random-effects models.

**Results:**

After deleting duplicates and closely evaluating all screened citations, we chose 60 studies with totally 5,960,224 participants for this analysis. Fourteen studies were included in the arrhythmia risk analysis, and 50 in the analysis of all-cause mortality. Hypoglycaemic patients had significantly higher risks of arrhythmia occurrence (RR 1.42, 95%CI 1.21-1.68), CVD-induced death (RR 1.59, 95% CI 1.24-2.04), and all-cause mortality (RR 1.68, 95% CI 1.49-1.90) compared to euglycaemic patients with significant heterogeneity.

**Conclusion:**

Hypoglycaemic individuals are more susceptible to develop cardiac arrhythmias and die, but evidence of potential causal linkages beyond statistical associations must await proof by additional specifically well planned research that controls for all potential remaining confounding factors.

## Introduction

Diabetes, a non-communicable disease, is a serious public health concern worldwide. More than half a billion people (536.6 million) aged 20-79 were predicted to develop diabetes mellitus (DM) in 2021, with roughly 90% having type 2 DM (T2DM) (1). Diabetes is a serious, chronic disease that profoundly affects the lives and well-being of individuals, families, and communities throughout the world. Diabetes is one of the leading ten causes of adult mortality, with a total estimated global healthcare expenditure of 966 billion USD (1).

Cardiovascular diseases (CVDs) are the most common cause of morbidity and mortality in this populace (2). Numerous large epidemiological studies demonstrate that maintaining target glycemic control is the only proven strategy for preventing diabetic vascular complications (3). To accomplish this goal, active use of oral diabetes medications or insulin as well as lifestyle modifications in accordance with the individual situation shall be initiated after the diagnosis of diabetes (4–6). However, intensive glycaemic control with antidiabetic drugs inevitably exposes IWD to the common side effect of hypoglycaemia (7–10). Recent evidence confirms that a hypoglycaemic episode, irrespective of severity, is clinically important because of its link to increased arrhythmias (via effects on cardiac repolarisation and alterations in cardiac autonomic activity, hypokalemia due to excess of insulin and increased secretion of catecholamines, which might drive potassium into the cell during hypoglycemia, and fuel energy shortage at the level of the cardiomyocyte due to low availability of glucose despite stress and increased demand as well (11, 12)) and other cardiovascular (CV) events and mortality (13–18). Similarly, a recent systematic review (19) reports that hypoglycaemia leads to electrocardiogram changes associated with a greater likelihood of cardiac arrhythmias, which are related to increased CV events and mortality. Another systematic review (20) concludes that hypoglycaemia is a risk factor for adverse vascular events and death. Nevertheless, contradictory evidence exists regarding the association between hypoglycaemia and the risks of cardiac arrhythmias and mortality (21–24). The aforementioned reviews have numerous limitations, including a lack of subgroup analysis, and a failure to recognise heterogeneity. Specifically, some undetected confounders (e.g. study design, hypoglycaemia criteria, ignorance of model adjustment, and dissimilar comorbidity profile) that are ignored by model adjustment can result in bias. Meanwhile, some studies (25) show that only spontaneous hypoglycaemia is associated with increased mortality, while others (26, 27) show no effect. Based on the available evidences, several authors (28, 29) even suggest that hypoglycaemia is a marker of disease severity and/or comorbidity burden in hospitalised patients.

For these reasons, our aim was to assess if hypoglycaemia affects the risk of arrhythmia or mortality in IWD.

## Methods

### Search strategy and criteria for inclusion

Two separate reviewers searched PubMed, EMBASE, Wiley Cochrane Central Register of Controlled Trials(CENTRAL), Web of knowledge, Scopus and CINAHL complete up to December 22, 2022 as per applicable recommendations (30). Any disagreement between them was resolved through consensus. In addition to keywords, we used Medical Subject Headings terms in PubMed, EMTREE terms in EMBASE, CINAHL headings in CINAHL, and keywords in all included databases as a part of our strategy. The search terms were “hypoglycaemia”, “hypoglycemia” or “hypogly*” and “diabetes” or “diabet*”, and “arrhythmias” or “arrhythmia*” or “dysrhythmia*” or “mortality” or “mortalit*” or “death” (search technique shown in Additional file 1). The grey literature was found by manually searching reference lists from eligible research and associated reviews. Authors were contacted to obtain necessary data. Prior to the start of this investigation, no review protocol for this systematic review has been published or registered.

The inclusion criteria were: (i) observational studies (OSs) [cohort, case-control, cross-sectional, and longitudinal studies], *post-hoc* analysis or sub-analysis of randomised controlled trials (p-h/sa of RCTs); (ii) focusing on the relationship of any hypoglycaemia with arrhythmias or CVD-induced death or overall death in IWD, and reporting risk or prevalence or incidence of arrhythmias (mortality) for IWD with hypoglycaemia as compared with euglycaemia, and (iii) standardized mortality or incidence ratio (SMR or SIR), or incidence rate ratio (IRR), or odds ratios (OR), or hazard ratio (HR), or relative risk (RR), as well as pertinent relevant raw data for recalculation are all used to report effect estimates. Exclusion criteria were as follows: (I) emphasizing the connection of hypoglycaemia with adverse outcomes such as arrhythmias- or CVD-caused death or all-cause mortality in non-diabetic people; (II) case report, quasiexperiment (in which subjects are not assigned at random), editorial, remark, review, letter or unpublished study; (III) only published as abstract or conference proceeding. Diabetes studies that did not provide such estimations were also omitted. When a site-specific dataset was published multiple times, the study with the newest t publication or the largest sample size was generally picked.

### Data extraction and quality evaluation

Data were collected about the research (design, first author’s name, title, year of publication, data source, country/region, baseline years, sample size, follow-up duration, diabetes clarity, endpoints, measure of relationship, number of observed and expected events), participants (mean age and the gender), analysis strategy (statistical models, adjustment factors), effect size (e.g., SMR or IRR, or SIR, or HRs, RRs or ORs), as well as pertinent raw data for recalculation.

Two researchers independently assessed the quality of each p-h/sa of RCT and enrolled study based on cohort or case-control design utilizing the 9-star Newcastle-Ottawa Scale (NOS) (31). A rating of above six stars (31) indicates high quality. The 11-item Agency for Healthcare Research and Quality (AHRQ) checklist is recommended for assessing the quality of cross-sectional or longitudinal studies (32, 33). Each item’s responses are “yes,” “no,” and “unclear”. If the response to each question is “yes”, one point is awarded; otherwise, no points are given. Studies with a total score of 0–5, 6–7, 8–11, respectively, were deemed to be of low, moderate and high quality. Disagreements were resolved through dialogue.

### Statistical analysis

The main and second goals were the hazards of arrhythmias and overall death (CVD death) whereas the analysis period, respectively. OR, or RR with 95% confidence interval (CI) was used to summarize dichotomous outcomes. ORs are comparable to RRs since the absolute risk of arrhythmias or death is minimal in these populations and the two indices provide equivalent RR estimates (34). The I^2^ statistic was used to determine the fraction of variability between studies because of heterogeneity between studies, and with I^2^ values greater than 50%, 25-50%, and less than 25%, was classified as high, medium, and low respectively (35). The iterative non-central Chi-2 test was used to find a CI for I^2^ (36).

Concerning the antecedent discrepancy of p-h/sa of RCT and OSs, we performed subgroup analyses for arrhythmia by sample size (<1000, and ≥1000), diurnal differences (nocturnal hypoglycaemia, and daytime hypoglycaemia), severity of hypoglycaemia (severe, and total), clarity of diabetes (T1DM, T2DM, and total), and type of arrhythmia (incidence of QTc interval prolongation, and other arrhythmia). For all-cause mortality analysis, subgroup analyses were conducted with sample size, country/region (developed, developing, and total), study population (simple diabetes, and diabetes with other disease or high risk for CV disease), hypoglycaemia episode (1, and ≥2), clarity of diabetes, study design (OSs, and p-h/sa of RCT), severity of hypoglycaemia, follow-up duration (≤1 year [including in-hospital mortality, and ICU mortality], and >1 year), and methods for effect estimate extraction (reported and calculated). Any quantifiable source of heterogeneity was identified through sensitivity analysis by excluding each study individually.

Publication bias was examined using Begg’s and Egger’s tests when at least five studies were available for analysis, as well as by visually inspecting the asymmetry of funnel plots of estimated effects against standard errors (37). Any publication bias (P<0.10) was corrected using Duval & Tweedie’s trim-and-fill approach. All other analyses were conducted using STATA 14.0 (US) at P < 0.05 significance level.

## Results

### Study identification

**Figure 1:**
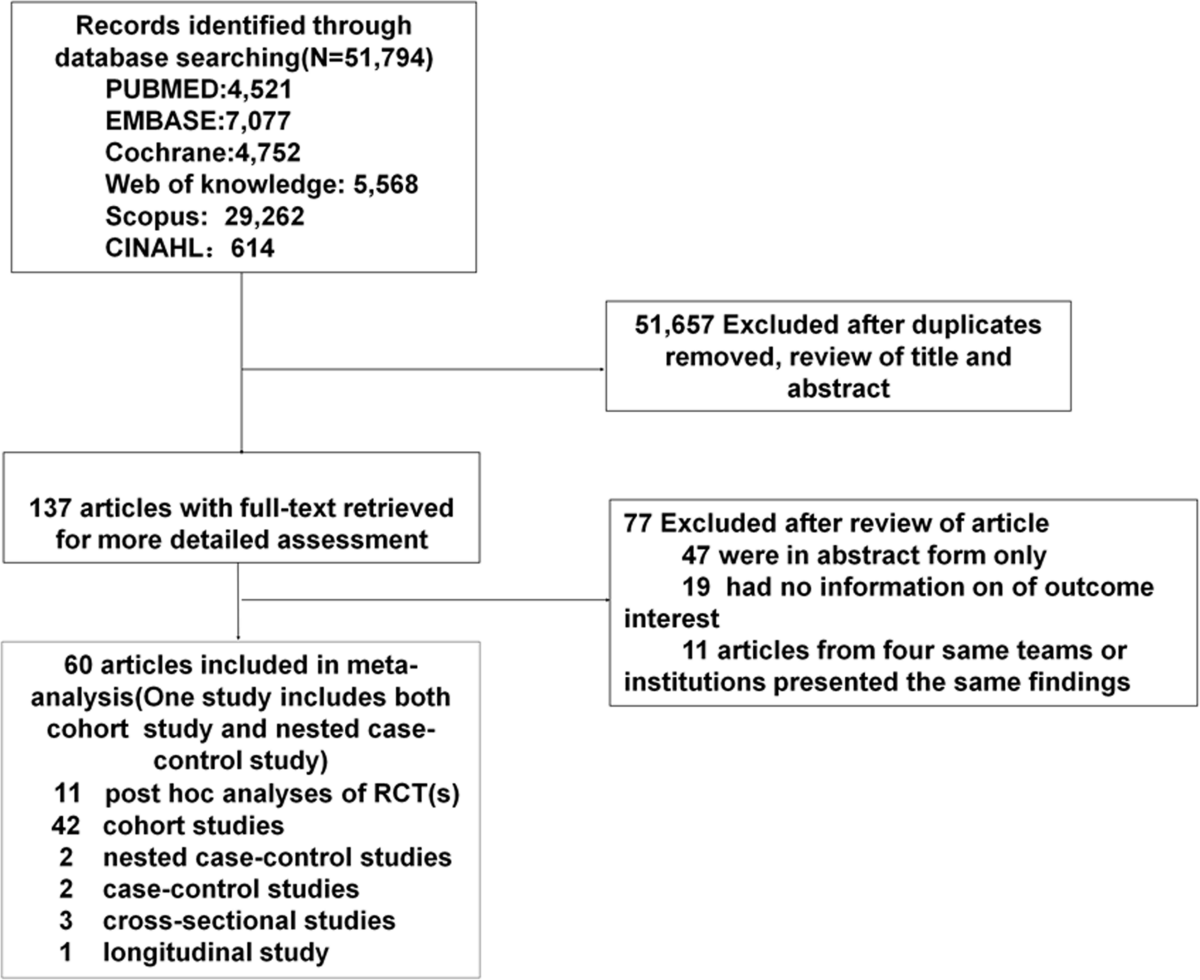
Flow diagram of study selection.

From the 51,794 articles discovered through a systematic search, 137 were selected for further analysis (**Figure 1**). Two articles (24, 38) from one data source presented conflicting findings, and thus were both included. Four studies (22, 39–41) each two from the same teams/institutions and each reporting different outcomes for analysis were included. Finally, 60 articles provided data on the relationship between hypoglycaemia and arrhythmias or mortality (**Table 1**). The 60 studies included 11 p-h/sa RCTs (13, 21, 22, 41, 51, 75–77, 82, 86, 91), and 50 OSs (41 cohort studies (14, 23, 24, 39, 40, 43–45, 47–50, 52, 53, 55–65, 67–70, 72, 73, 78–81, 83, 85, 87–90) 3 (nested) case-control studies (54, 71, 84), 3 cross-sectional studies (38, 42, 74) 1 longitudinal study (66) and one study (46) including both cohort and nested case-control studies). The sample sizes ranged from 25 to 2,032,689 patients, and the mean age of patients ranged between 25.6 and 81.86 years. The female proportion ranged from 2.90% to 59.27%, and duration of DM was from 1.36 to 44.25 years.

**Table 1:** Detailed characteristics of studies included in the meta-analysis.

Study	Data source /Country; Region	Study design	Population	Sample Size	Baseline years/Duration of follow-up	Mean age (years)	Female (%Total)	Mean DM duration(years)	Severity of hypoglycemia	Hypoglycaemia definition^a^	Outcomes assessment:/ Effect Estimate
GRUDEN et al. (38)	EURODIAB Prospective Complications Study /Multicenter from 16 European countries	Cross-sectional	T1DM	3,248	NR/NA	NR	NR	14.7	Severe	1	Incidence of QTc interval prolongation/OR
Pistrosch et al. (42)	One outpatient department for metabolic diseases/Germany	Cross-sectional	T2DM with a proven cardiovascular event	94	2012-2014/5 days	67.68	21.28	17.07	Any	6	Ventricular tachycardia/RR(calc)
Chow et al. (23)	Sheffield Teaching Hospitals diabetes outpatient clinics /The United Kindom	Cohort	T2DM	25	NR/5 days	64	48.0	17.0	Any	5	Bradycardia, Atrial ectopic, VPB, Complex VPB/IRR
Tsujimoto et al. (43)	The National Center for Global Health and Medicine/Japan	Cohort	Total	192	2006-2012/NR	72.06	33.87	15.17	Severe	1	Incidence of QTc interval prolongation/aOR
Amione et al. (24)	EURODIAB Prospective Complications Study/Multicenter from 16 European countries	Cohort	T1DM	1,415	1989-1991/7.0 years	32.1	NR	14.2	Severe	1	Incidence of QTc interval prolongation/aOR
Ko et al. (44)	The Korean NHIS/Korea	Cohort	T2DM	1,509, 280	2005-2008/8.6 years	54.9	36.2	NR	Severe	1	AF, All-cause mortality/aHR
Novodvorsky et al. (45)	Sheffield Teaching Hospitals outpatient clinics/The United Kindom	Cohort	T1DM	37	NR/96 hours	34.0	48.6	19.3	Any	5	Bradycardia, Atrial ectopic, VPB/IRR
Lee et al. (39)	The ARIC study/The United States	Cohort	T2DM	1,209	1996-1998/3.0 years	63.60	54.37	NR	Severe	3	AF, All-cause mortality, CVD mortality/aHR
Zhang et al (46)	UEBMI claims of Tianjin/China	Cohort; Nested case-control	T2DM	8,466	2008-2015/2.58 years	58.97	47.64	NR	Severe	1	Arrhythmias, All-cause mortality/aHR, aOR
Mylona et al. (47)	A multicentre study from eight hospitals (nine clinics)/Greece	Cohort	Total	249	NR/16 months	72.25	50.20	15.06	Any	8	Incidence of QTc interval prolongation/RR(calc)
Echouffo-Tcheugui et al. (40)	The ARIC study/The United States	Cohort	T2DM	2,193	2011-2013/6.1 years	75.85	57.27	10.44	Severe	3	AF, All-cause mortality /aHR
Abdelhamid et al. (48)	Mixed medical-surgical ICUs in two geographically distinct university-affiliated hospitals/Australia, The United Kindom	Cohort	T2DM	31	2016-2019/5 days	65.0	39.0	21.16	Any	5	Bradycardia, Atrial ectopic, VPBs/IRR
Andersen et al. (49)	The diabetes outpatient clinics at Steno Diabetes Center Copenhagen, and Nordsj.llands Hospital Hillerod/Denmark	Cohort	T2DM	21	NR/1.0 years	66.8	28.6	18.2	Any	4	Arrhythmia/IRR
Kaze et al. (41)	ACCORD study/The United States, Canada	*Post-hoc* analyses	T2DM	8,277	NR/5.0 years	62.6	38.7	9.0	Severe	2	Incidence of QTc interval prolongation/aRR
Mellbin et al. (21)	DIGAMI 2 trial/Sweden	*Post-hoc* analyses	T2DM with AMI	1,253	1998-2003/2.1 years	68.39	33.2	7.90	Any/severe	6/11	All-cause mortality, CVD mortality/aHR
Curkendall et al. (50)	HIPAA-compliant operating policies and procedures/The United States	Cohort	Total	107,736	2000-2006/NR	65.93	51.25	NR	Any/Severe	4/7	In-hospital mortality/aOR
Bonds et al. (22)	ACCORD study/The United States	*Post-hoc* analyses	T2DM	10194	NR/3.5 years	NR	NR	NR	Symptomatic severe	2	All-cause mortalit/aHR
Zoungas et al. (51)	ADVANCE study /Multicenter from 20 countries	*Post-hoc* analyses	T2DM	11,140	2001-2003/3 months, 6 months,5.0 years	NR	NR	NR	Severe/Minor	2/10	All-cause mortality, CVD mortality/aHR
Li et al. (52)	The Department of Cardiology, Xuanwu Hospital, Capital Medical University/China	Cohort	Total with AMI	246	1995-2005/NR	NR	NR	NR	Any	8	In-hospital mortality/RR(calc)
Nirantharakumar et al. (53)	University Hospital Birmingham/The United Kindom	Cohort	Total	6,374	2007–2010/NR	66.30	42.48	NR	Mild~moderate/Severe	4/7	Inpatient mortality/RR(calc)
MCCOY et al. (54)	Mayo diabetes clinic/The United States	Case-control	Total	1,013	2005-2006/5.0 years	60.5	45.2	1.36	Mild/Severe	9/1	All-cause mortality/aOR
ZHAO et al. (55)	VISN 16/The United States	Cohort	T2DM	1,522	2004-2010/3.93 years	62.58	3.9	NR	Any	3	All-cause mortality /aHR
ORIGIN Trial Investigators et al. (13)	ORIGIN Trial/Multicenter from 40 countries	*Post-hoc* analyses	Total	12,537	2003-2005/6.2 years	63.53	34.98	NR	Severe	7	All-cause mortality, CVD mortality/aHR
Tan et al. (56)	Derriford Hospital medical assessment unit/The United Kindom	Cohort	Total	1,457	2010 -2011/NR	71.0	NR	NR	Any	4	In-hospital mortality/RR(calc)
HSU et al. (14)	The NHIRD/Taiwan	Cohort	T2DM	9,220	1998–2009/11.0 years	63.30	56.29	4.96	Mild/Severe	The outpatient/hospital claims dataset	All-cause mortality/HR
Sechterberger et al. (57)	a 24-bed mixed surgical/medical ICU in a teaching hospital/Netherlands	Cohort	Total	1,638	2004-2011/NR	68.0	37.0	NR	Any	7	ICU mortality/aOR
Cooper et al. (58)	the Western Australia Children’s Diabetes Database/Australia	Cohort	T1DM	1,309	1987-2012/7.6 years	25.6	49.60	NR	Severe	1	All-cause mortality/aHR
Lee et al. (59)	The KAMIR and the KorMI/Korea	Cohort	T2DM with AMI	20,714	2005–2008, 2008–2012/30 days	64.09	37.25	NR	Any	4	30-day mortality/aHR
Kong et al. (60)	Hong Kong Diabetes Registry/Hong Kong, China	Cohort	T2DM with or without CKD	8,767	1995- 2007/6.66 years	58.46	53.02	5.49	Severe	7	All-cause mortality /aHR
Lung et al. (61)	The Swedish National Diabetes Register/Sweden	Cohort	T1DM with a major cardiovascular complication	1,839	2002–2010/28 days	59.46	45.0	44.25	Any	3	All-cause mortality/HR, OR
Elwen et al. (62)	an emergency services call-out for hypoglycemia/The United Kindom	Cohort	Total	1,156	2005-2013/12 months	61.0	40.0	NR	Any	4	All-cause mortality /OR
Khunti et al. (63)	The CPRD database/The United Kindom; the HES data/England	Cohort	T1DM/T2DM with or without History of CVD	13,682	2001-2007/5 years	62.28	44.0	4.85	Severe or nonsevere /Severe	7	All-cause mortality /aHR
Gómez-Huelgas et al. (64)	The BMDS registry/Spain	Cohort	Total	309,008	1997-2010/NR	72.0	49.1	NR	Any	3	In-hospital mortality /OR
Escalada et al. (65)	Medicare Advantage claims database/The United States	Cohort	T2DM	31,035	2007-2012/2.9 years	72.0	53.0	NR	Any	1	All-cause mortality, CVD mortality /HR
Freemantle et al. (66)	The CREDIT study/Multicenter from 12 countries(10 in Europe, one in Canada and one in Japan)	Longitudinal	T2DM	2,999	NR/54 months	61.0	48.8	9.0	Any/Severe	4/1	All-cause mortality /aHR
Rauh et al. (67)	the Hoorn Diabetes Care System Cohort/Netherlands	Cohort	T2DM	1,667	2000-2002/1.9 years	67.2	47.0	11.5	Mild/Severe	11/1	All-cause mortality /aOR
Takeishi et al. (68)	Ichinomiyanishi Hospital/Japan	Cohort	Total with various infections	620	2009- 2014/5 years	79.0	39.19	NR	Any	4	In-hospital mortality /aOR
Cha et al. (69)	VDR/Korea	Cohort	T2DM	906	2000 -2010/10.4 years	55.76	59.27	8.17	Severe	1	All-cause mortality, CVD mortality/aHR
Sejling et al. (70)	Nordsjællands Hospital Hillerød/Denmark; The Radboud University Medical Centre/Netherlands	Cohort	T1DM	751	Denmark: 1999–2001/12 years; Netherlands: 2006–2008/6.5 years	45.61	49.04	23.85	Severe	1	All-cause mortality, CVD mortality/HR
Lu et al. (71)	Taiwan’s NHI research database/Taiwan	nested case-control	T1DM	10,314	1997- 2011/<1 year, 3-5 years	41.42	42.18	NR	Severe	1	All-cause mortality /aOR
Chevalier et al. (72)	IMS HDD/Belgium	Cohort	Total	30,710	2011-2014/NR	NR	NR	NR	Any	3	In-hospital mortality /aOR
CHI et al. (73)	Taiwan’s NHI research database/Taiwan	Cohort	Total	10,623	2001-2011/7.0 years	74.1	49.2	NR	Any	3	All-cause mortality /aRR
Zhao et al. (74)	the China PEACE−Retrospective AMI study/China	Cross-sectional	Total with AMI	2,280	2001, 2006, and 2011/NR	NR	NR	NR	Any	4	In-hospital mortality /aOR
Standl et al. (75)	TECOS/Multicenter from 38 countries	*Post-hoc* analyses	T2DM	14,671	2008-2012/3.0 years	65.04	29.29	10.08	Severe	1	All-cause mortality, CVD mortality/aHR
Zinman et al. (76)	LEADER cardiovascular (CV) outcomes trial /Multicenter from 32 countries	*Post-hoc* analyses	T2DM with high risk for CV disease	9,340	2010-2012/3.8 years	64.26	36.37	12.79	Any	6	All-cause mortality, CVD mortality/aHR
Pieber et al. (77)	DEVOTE 3/Multicenter from 20 countries	*Post-hoc* analyses	T2DM with high risk of cardiovascular events	7,637	2013-2014/≤1 year	64.97	37.44	16.43	Severe	1	All-cause mortality/HR
Leung et al. (78)	MacKay Memorial Hospital/Taiwan	Cohort	Total with carbapenem-resistant Acinetobacter baumannii complex bacteremia	146	2010-2015/5 years	69.23	41.1	NR	Any	4	All-cause mortality /RR(calc)
Lo et al. (79)	NHIRD/Taiwan	Cohort	T1DM/T2DM	30,471	1999-2001/≤1 year; ≥1 year	64.63	48.80	NR	Severe	3	All-cause mortality /aHR
Wei et al. (80)	The Central Hospital of Wuhan/China	Cohort	T2DM	1,520	2013-2017/31 months	59.43	48.49	6.79	Any	4	All-cause mortality, CVD mortality/aHR
Yun et al. (81)	The Korean NHI System database/Korea	Cohort	T2DM	1,568,097	2007-2009/≤12months; >24-months	58.60	45.30	NR	Severe	3	All-cause mortality/aHR
Davis et al. (82)	VADT/The United States	Post -hoc analysis	T2DM	1,791	2000-2000/3 months	60.40	2.90	11.5	Severe	1	All-cause mortality, CVD mortality/aHR
Wernly et al. (83)	The Jena University Hospital/German	Cohort	T2DM	685	2004 -2009/4-7 years	70.11	NR	NR	Any	4	Intra-ICU mortality, Long-term mortality/OR, HR
Ferreira et al. (84)	Centro Hospitalar do Porto/Portugal	Case-control	Total with community-acquired pneumonia or chronic obstructive pulmonary disease	242	2016-2016/NR	77.0	42.1	NR	Any	4	In-hospital mortality/RR(calc)
Mattishent et al. (85)	The CPRD database/The United Kindom	Cohort	Total with or without dementia	19,993	1997-2016/1 year, 12-60months	78.88	53.0	NR	Any	7	All-cause mortality /aHR
Standl et al. (86)	EXSCEL/Multicenter from 35 countries	Post -hoc analysis	T2DM	14,752	2010-2015/3.2 years	62.03	37.98	12.06	Severe	1	All-cause mortality, CVD mortality /aHR
Jensen et al. (87)	The Danish National Patient Register or the National Pharmacological Database/Denmark	Cohort	T1DM/T2DM	27,746	1996 - 2017/T1DM:8.9-year;T2DM:2.3-year	57.49	41.66	11.81	Severe	3	All-cause mortality/aHR
Zaccardi et al. (88)	The CPRD database/The United Kindom	Cohort	T2DM	74,610	1998- 2011/7.1 years	67.73	45.22	NR	Severe	1	All-cause mortality, CVD mortality/RR(calc)
Han et al. (89)	The Korean NHIS/Korea	Cohort	T2DM with or without dementia	2,032,689	2009-2015/6.9 years	59.85	42.33	6.89	Severe	1	All-cause mortality /aHR
Cha et al. (90)	St. Vincent’s Hospital/Korea	Cohort	T2DM with heart failure	397	2016-2018/25 months	73.08	52.64	12.00	Any	4	All-cause mortality, CVD mortality /RR(calc)
Heller et al. (91)	LEADER study/Multicenter from 32 countries	*Post-hoc* analyses	T2DM	9,340	2010-2012/3.8 years	64.30	35.70	12.80	Any/Severe	6	All-cause mortality, CVD mortality /RR(calc)

DM, diabetes mellitus; T1DM, Type 1 diabetes; T2DM, Type 2 diabetes; CVD, Cardiovascular disease; QTc, corrected QT interval; VPB, Ventricular Premature Beats; AF, Atrial fibrillation; AMI, Acute myocardial infarction; NHI, National Health Insurance; NHIS, National Health Insurance Service; ARIC, Atherosclerosis Risk in Communities; UEBMI, Urban Employee Basic Medical Insurance; ACCORD, Action to Control Cardiovascular Risk in Diabetes; DIGAMI 2,The second Diabetes Glucose and Myocardial Infarction; HIPAA, Health Insurance Portability and Accountability Act; ADVANCE, Action in Diabetes and Vascular Disease: Preterax and Diamicron Modified Release Controlled Evaluation; ORIGIN, Outcomes Reduction with an Initial Glargine Intervention; NHIRD, The National Health Insurance Research Database released; KAMIR, The Korea Acute Myocardial Infarction Registry; KorMI, The Korea Working Group on Myocardial Infarction; CPRD, The Clinical Practice Research Datalink; HES, Hospital Episode Statistics; BMDS, The Basic Minimum Data Set; VDR, The Vincent Type 2 Diabetes Registry; HDD, Hospital Disease Database; LEADER, Liraglutide Effect and Action in Diabetes: Evaluation of Cardiovascular Outcome Results; DEVOTE, Trial Comparing Cardiovascular Safety of Insulin Degludec vs Insulin Glargine in Patients with Type 2 Diabetes at High Risk of Cardiovascular Even; NHIRD, The National Health Insurance Research Database; CPRD, The Clinical Practice Research Datalink; VADT, The Veterans Affairs Diabetes Trial; VISN 16, Veterans Integrated Service Network 16; CREDIT, The Cardiovascular Risk Evaluation in people with type 2 Diabetes on Insulin Therapy; PEACE−Retrospective AMI, Patient−centered Evaluative Assessment of Cardiac Events−Retrospective acute myocardial infarction study; TECOS, Trial Evaluating Cardiovascular Outcomes With Sitagliptin; EXCEL, Exenatide Study of Cardiovascular Event Lowering; IG, interstitial glucose; HR, Hazard ratio; OR, odds ratio; IRR, incident rate ratio; aHR, Adjusted hazard ratio; aOR, Adjusted odds ratio; aRR, Adjusted risk ratio; NR, not reported; NA, not appliable.

a^1^ denoted as an attack serious enough to require the help of another person or an episode resulting in hospital admission or loss of consciousness; a^2^ denoted as either a blood glucose concentration <2.7~2.8mmol/l (<50 mg/dl) or symptoms that resolved with treatment and that required either the assistance of another person or medical assistance; a^3^ denoted as definition with ICD-9 codes/ICD-10 codes/Read codes; a^4^ denoted as a blood glucose concentration ≤ 3.9~4.0 mmol per liter (70~72 mg per deciliter); a^5^ denoted as a blood glucose concentration ≤ 3.5 mmol per liter (63 mg per deciliter); a^6^ denoted as a blood glucose concentration ≤ 3.0 mmol per liter (54 mg per deciliter); a^7^ denoted as a blood glucose concentration < 2.0~2.8 mmol per liter (36~50 mg per deciliter) or the presence of typical symptoms and signs of hypoglycemia without other apparent cause; a^8^ denoted as a blood glucose concentration ≤ 5 mmol per liter (70 mg per deciliter); a^9^ denoted as symptoms of dizziness, blurry vision, confusion, and/or sweating that the patient was able to terminate without assistance; a^10^ denoted as either a blood glucose concentration < 2.0~2.8 mmol per liter (36~50 mg per deciliter) or symptoms that resolved with themselves; a^11^ denoted as either a blood glucose concentration ≤ 3.0 mmol per liter (54 mg per deciliter) or the presence of typical symptoms.

Apart from 10 studies on multinational origins, other origins were mentioned developed countries or regions in 46 articles, and developing countries in 4 articles. More than 50% studies (34/60) reported severe hypoglycaemia. Sixteen articles reported IWD with comorbidities or at high risk of CVD. Definitions of hypoglycaemia varied widely and were mostly based on blood glucose concentration or the presence of symptoms necessitating the assistance of another individual or medical help. Ten, 46, and 15 articles presented data on arrhythmia risk only, all-cause mortality only, and CVD death only, respectively, and four studies reported data on both arrhythmia and all-cause mortality risk.

### Quality evaluation

For cardiac arrhythmia analysis, quality analysis showed about 7 of 11 cohort studies and one case-control study were of high methodological quality, with ≥7 NOS scores (mean= 7.58; **Table S1**). One p-h/sa of RCT showed high quality according to NOS scores. Two cross-sectional studies were of moderate quality according to AHRQ (**Table S1**). When analysing the risk of all-cause mortality in 46 studies, 24 cohort studies and 2 case-control studies were of high methodological quality, with ≥7 NOS scores (mean= 7.73; **Table S1**). According to AHRQ, one cross-sectional study and one longitudinal research were of moderate quality. All 10 p-h/sas of RCTs were of high quality (mean NOS score= 8.50; **Table S1**).

### Risk of cardiac arrhythmia

**Figure 2:**
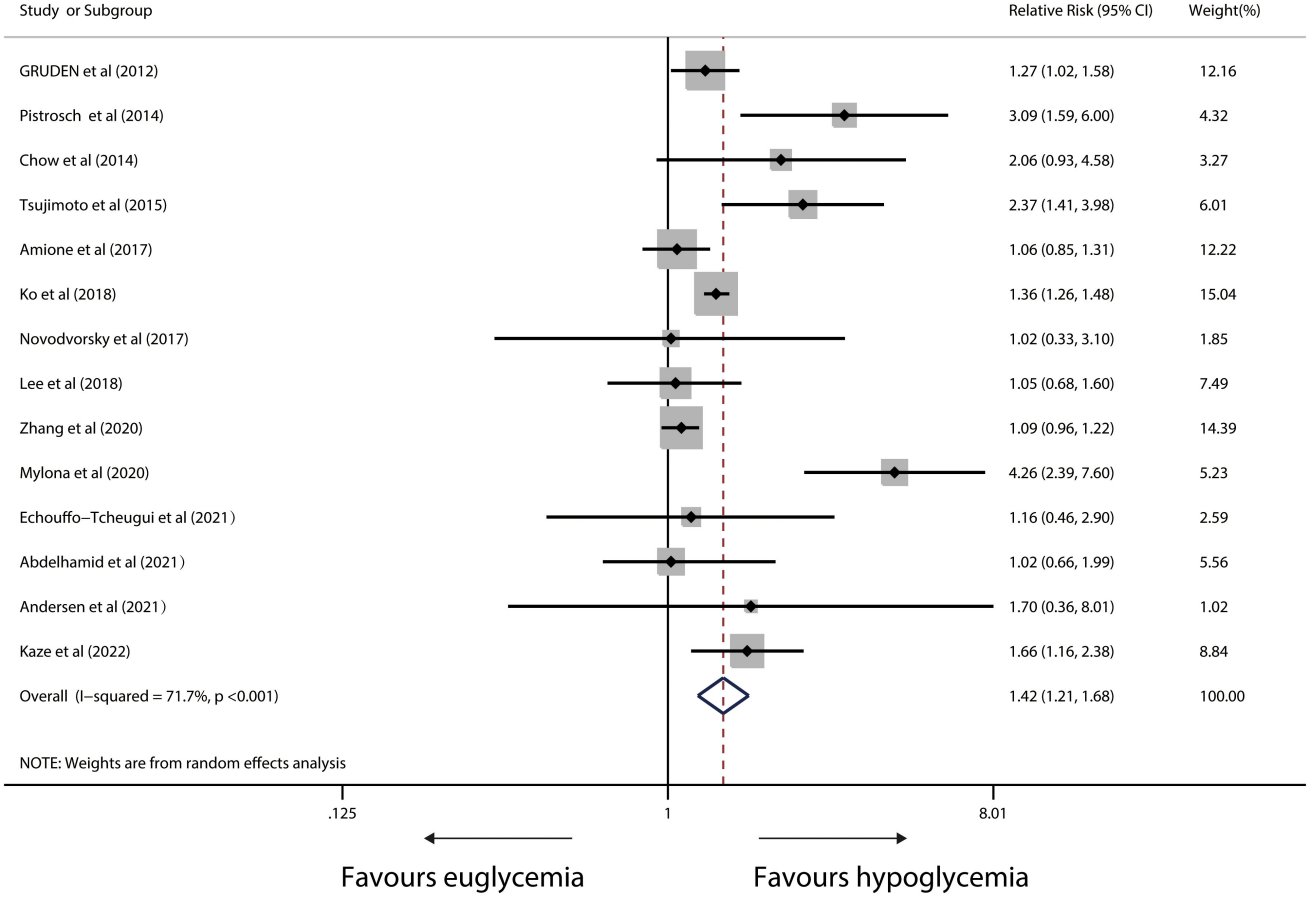
Forest plot for the association between hypoglycaemia with risk of cardiac arrhythmia in diabetic patients (X-axis: log scale; solid square: relative risk; horizontal lines: 95% CIs. The same in other figures).

The analysis of the connection between hypoglycemia and the risk of arrhythmia involved fourteen relevant studies. Overall, the pooled RR showed a 42% greater incidence of cardiac arrhythmia in IWD with versus without hypoglycemia (RR,1.42; 95%CI,1.21-1.68; p<0.001), but there was evident heterogeneity between studies (I^2^ = 71.7%, p<0.001) (**Figure 2**). Sensitivity analysis revealed that heterogeneity did not vanish after single studies were removed. In the arrhythmia risk analysis, Funnel plots revealed no evidence of systematic bias (Begg’s test, P=0.443; Egger’s test, P=0.245) (**Figure S1**).

**Figure 3:**
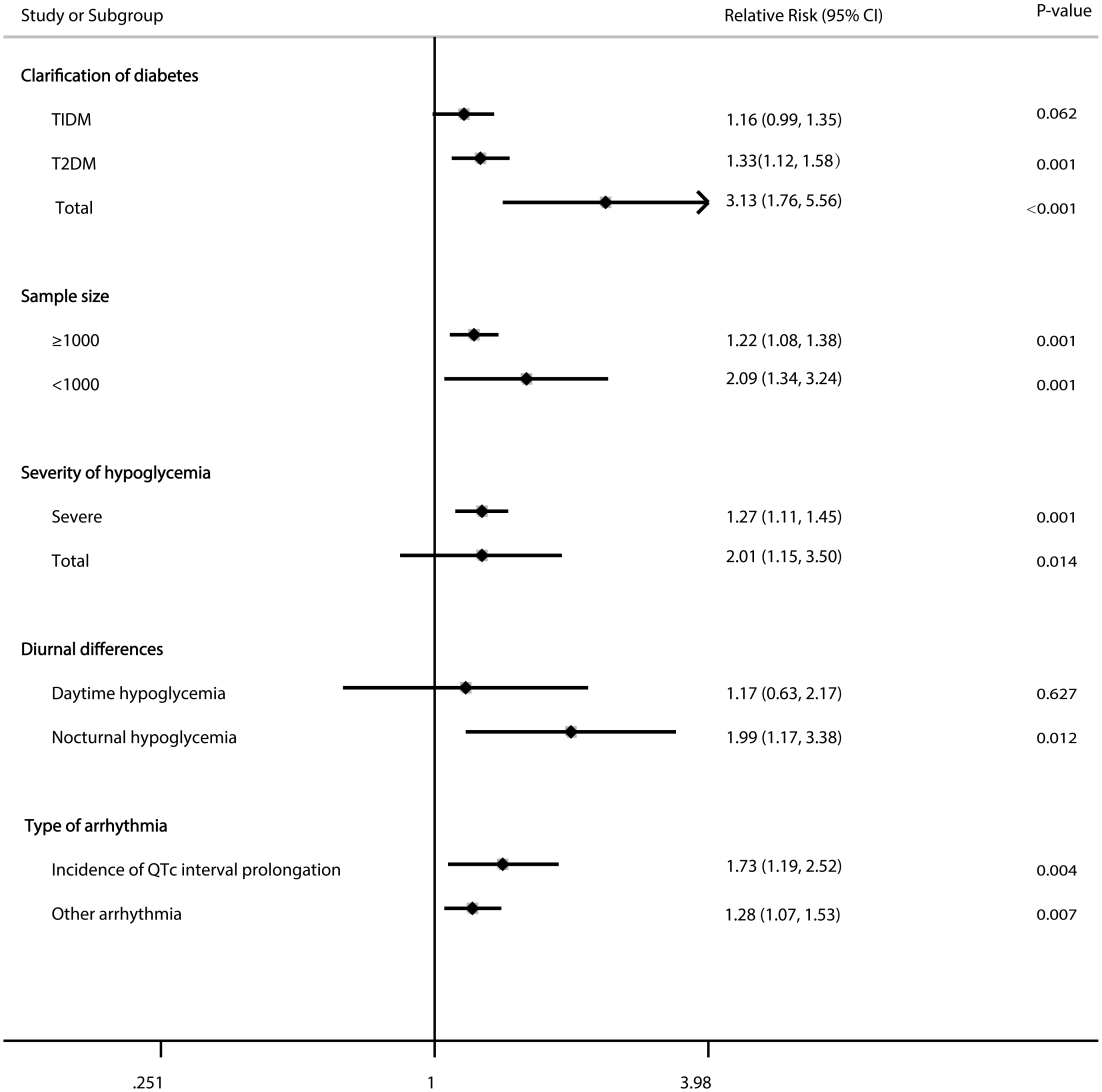
Forest plot for the association between hypoglycaemia with risk of cardiac arrhythmia in diabetic patients according to some clinically important variables.

The pooled RRs were essentially uniform regardless of the sample size (<1000 patients, P=0.001; ≥1000 patients, P=0.001), severity of hypoglycaemia (severe hypoglycaemia P=0.001; total hypoglycaemia, p=0.014), noctural hypoglycaemia (P=0.012), type of arrhythmia (incidence of QTc interval prolongation, P=0.004; other arrhythmia, P=0.007), T2DM (P =0.001), or total diabetes (P<0.001) (**Figure 3**).

### Risk of all-cause mortality

**Figure 4:**
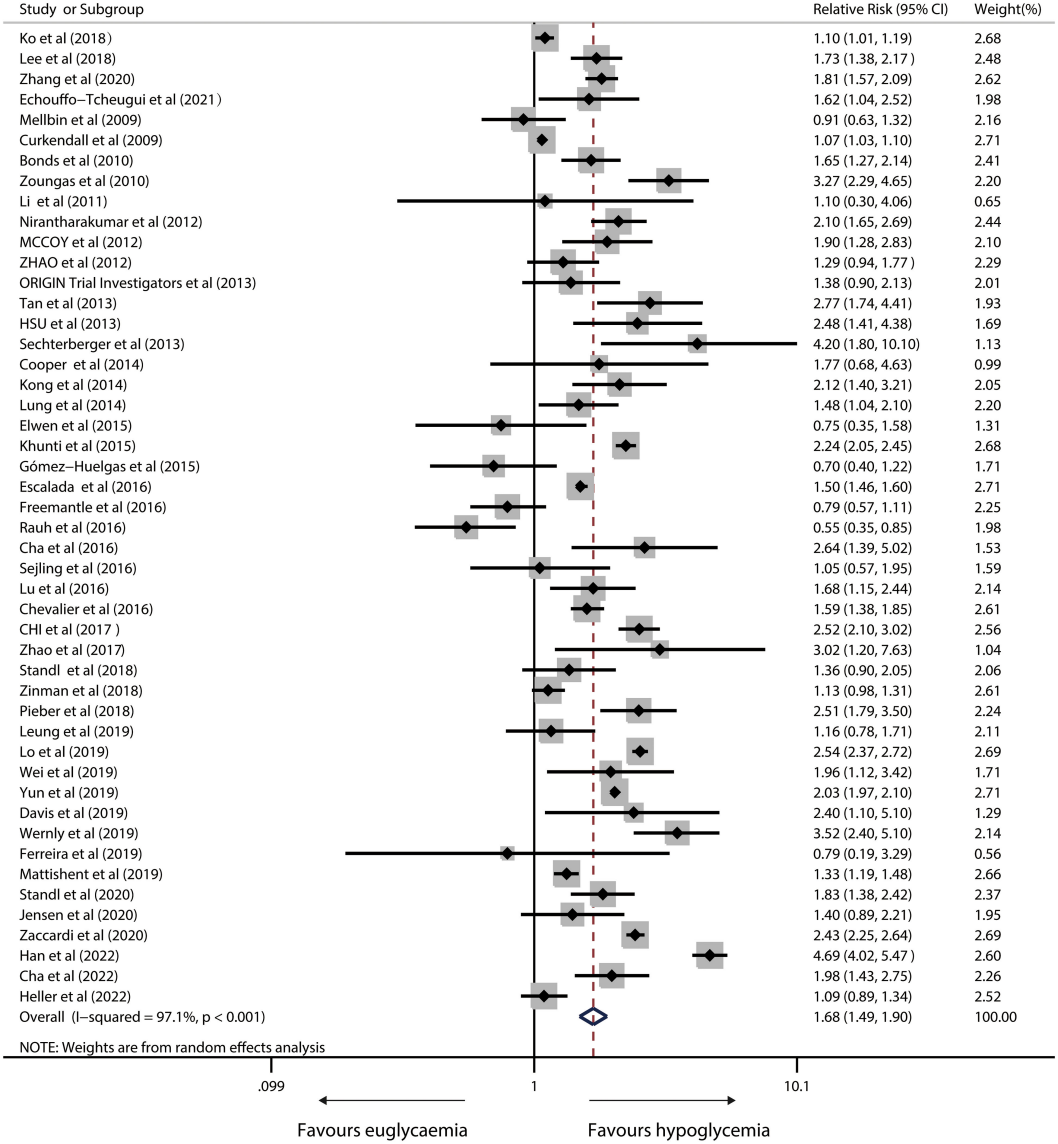
Forest plot for the association between hypoglycaemia with risk of all-cause mortality in diabetic patients.

Fifty studies reported a link between hypoglycemia and overall death in IWD. In a random-effects model, the pooled RR was 1.68 (95%CI, 1.49 to 1.90; P<0.001) with severe heterogeneity (I^2^ = 97.1%; P<0.001; **Figure 4**).

A sensitivity analysis revealed that heterogeneity did not vanish after removing single studies. Neither Egger’s test (P = 0.514) nor visual inspection revealed significant publication bias (**Figure S2**).

**Figure 5:**
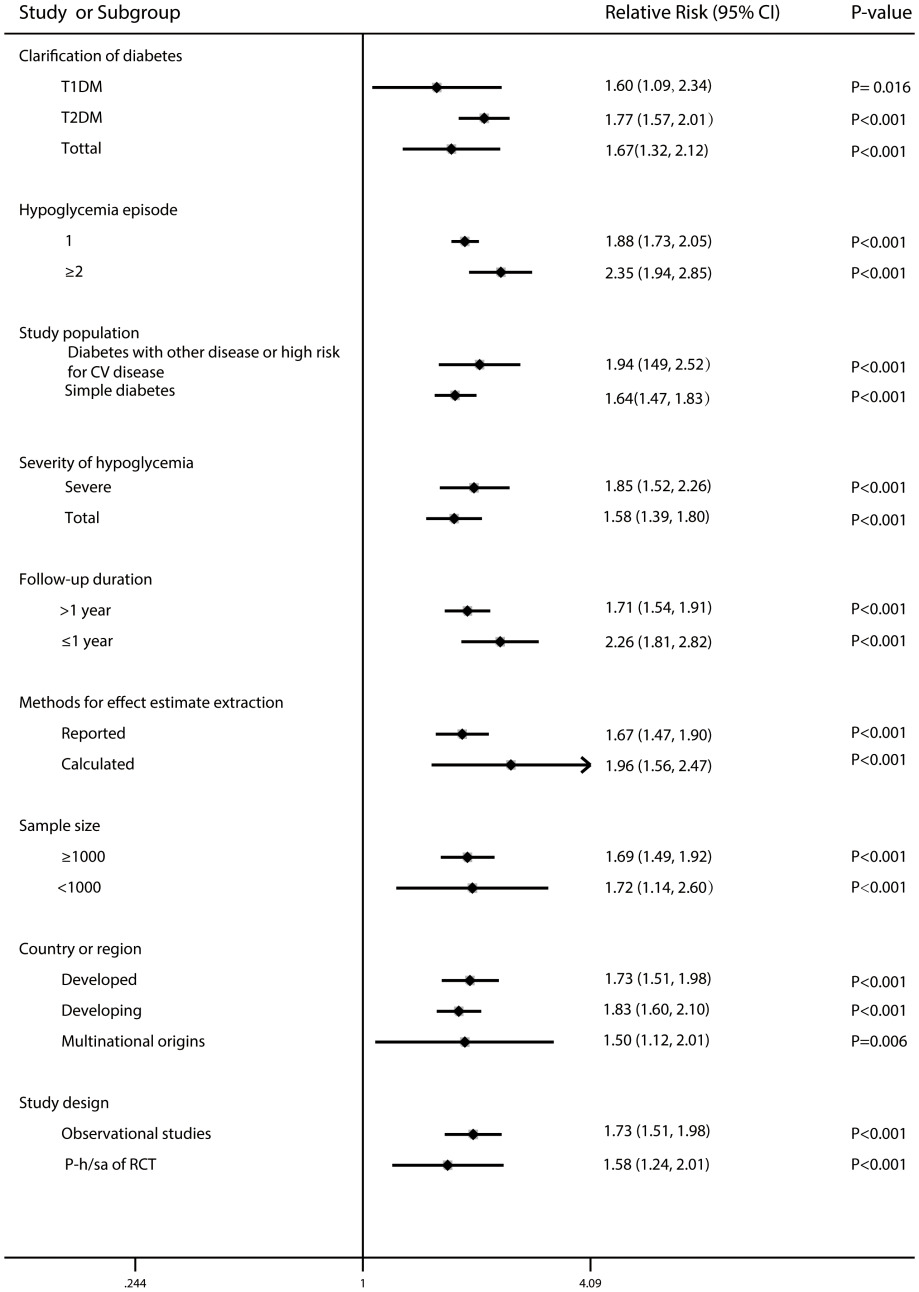
Forest plot for the association between hypoglycaemia with risk of all-cause mortality in diabetic patients according to some clinically important variables.

Estimated sample size, follow-up duration, country/region, study population, clarity of diabetes, study design, severity of hypoglycaemia, hypoglycaemia episode and methodologies for effect estimate extraction yielded similar results (**Figure 5**).

### Risk of CVD death

**Figure 6:**
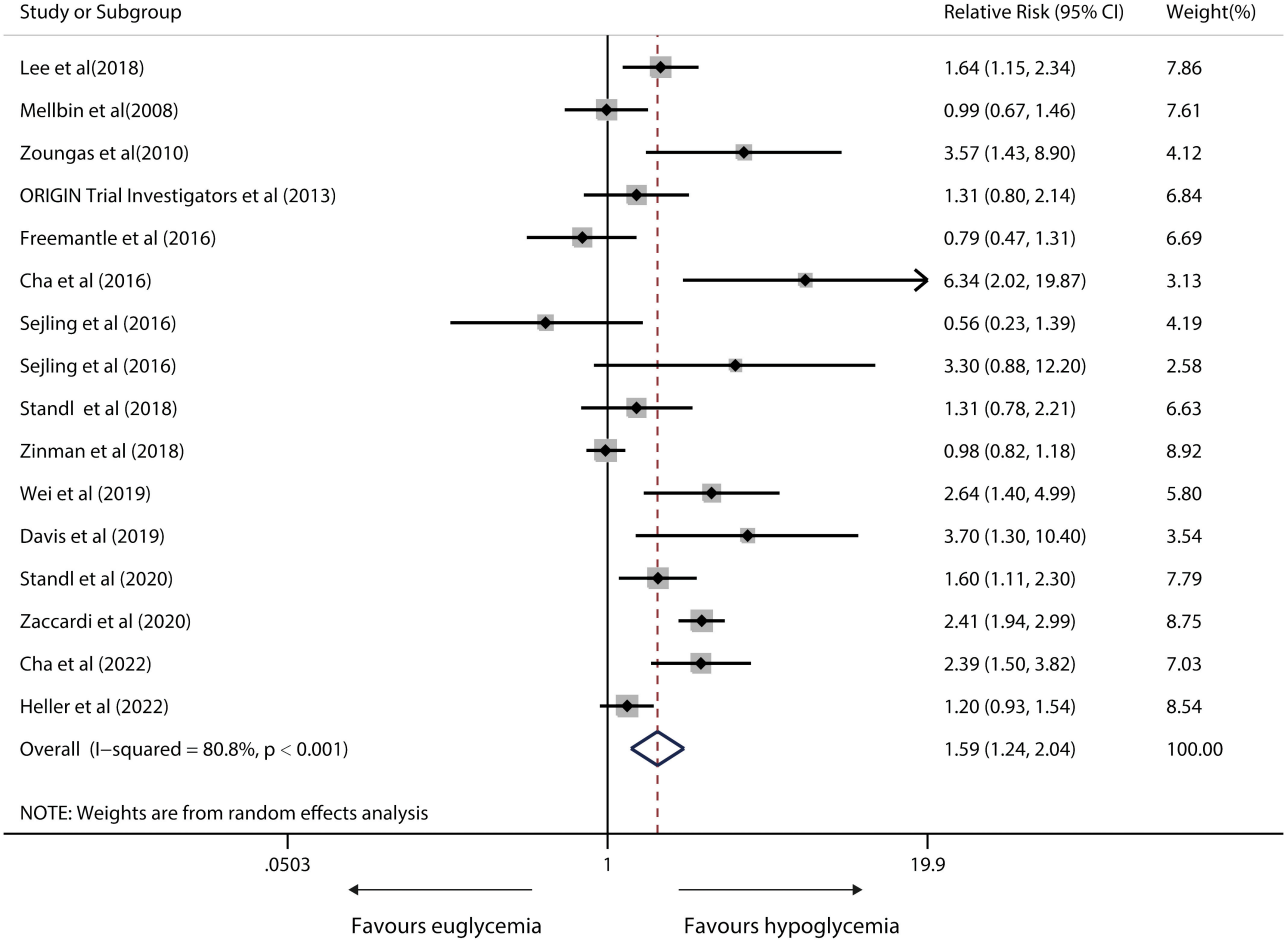
Forest plot for the association between hypoglycaemia with risk of CVD death in diabetic patients according to some clinically important variables.

A random-effects model exhibiting substantial heterogeneity (I^2^= 80.8%; P<0.001) from 15 relevant studies found that hypoglycemic versus euglycaemic IWD had a 59% significantly higher risk (pooled RR, 1.59; 95%CI, 1.24-2.04; P<0.001) in susceptibility to CVD-caused death. **Figure 6** depicts meta-analysis forest plots. A sensitivity analysis revealed that heterogeneity did not vanish after single studies were removed. Tests by Begg’s and Egger’s tests showed no clear systematic bias in the CVD-induced death risk analyses (both P > 0.1; **Figure S3**).

## Discussion

All current studies with 5,960,224 individuals were reviewed to find out evidence of a link between hypoglycemia and negative consequences, including cardiac arrhythmia, overall death, and CVD-induced death. We did not aim to establish a direct relationship, but did highlight the importance of hypoglycemia in relation to CV events and death. It is generally recognised that hypoglycemia, particularly severe hypoglycemia (SH), carries a greater proarrhythmic risk than euglycemia or hyperglycemia, as also observed in this study. The proarrhythmic mechanisms of hypoglycaemia, especially SH, may be various (92). Low glucose directly affects the human Ether-à-go-go Related Gene ion channel (93). Hypokalemia, and catecholamine both delay cardiac repolarisation, increasing the risk of early afterdepolarisations and ventricular arrhythmias. QT prolongation throughout the day may further trigger early afterdepolarisations. In the same way, activation of the sympathetic nervous system and an increase in calcium in the cytosol can lead to delayed afterdepolarisation and early heartbeats, which contribute to more ventricular ectopic activity during the day. Nevertheless, cardiac arrhythmias induced by hypoglycemia exhibit diurnal changes, which were also discovered in our study by subgroup analyses. Arrhythmia rates are even higher at night or early morning, when sympathoadrenal responses are suppressed (94). A sluggish sinus rate at a state of vagal dominance may disclose latent pacemakers (especially under situations of heightened automaticity), which causes excessive atrial and ventricular ectopic activity during nocturnal hypoglycaemia (45).

Even though the overall pooled RR suggests a 42% substantially higher likelihood of cardiac arrhythmia in hypoglycemic patients over euglycaemia patients, around 57% (8/14) recruited studies report no significant link between hypoglycemia and cardiac arrhythmia. There can be several reasons for this null result. First, the antagonistic autonomic nerve responses are a fundamental mechanism underlying the harmful effect of SH, but can be diminished in patients with long-standing diabetes, long follow-up, or established cardiovascular risks (95–97). Considering the involved patients here have relatively longer follow-up time, the effect of hypoglycemia is most likely obscured by the highly frequent cardiovascular and cerebrovascular comorbidities at baseline. Amione et al. (24) investigated the possible impact of repeated bouts of SH as a marker of QTc prolonged QTc interval over a seven-year period, and concluded that the impact of prediction could be due to adaptive mechanisms (98, 99). Second, earlier research revealed a dose-response or linear relationship between hypoglycemia and various unfavorable outcomes (71, 100), though only two studies (24, 41) were included. Third, individuals with repeated hypoglycemic episodes typically have a diminished awareness of hypoglycemia (101), which explains why there is no apparent temporal link between hypoglycemia and arrhythmia.

As for the mortality analysis, we did not aim to prove causality, but did highlight the associations between hypoglycemia and death. Due to pre-existing comorbidities, CVD-induced death usually accounted for the majority of deaths in the diabetes group. The relationship between hypoglycaemia and cardiovascular events is bidirectional in accordance with previous studies (75, 86). Firstly, we discovered a link between hypoglycemia and all-cause mortality in IWD, with a higher all-cause mortality in individuals with hypoglycemia compared to those without. The underlying biological mechanisms directly linking hypoglycaemia to death have yet to be elucidated. Nevertheless, some established scientific hypotheses suggest the fundamental mechanisms are primarily sympathoadrenal activation, cardiac or cerebral ischemia, abnormal cardiac repolarisation, QT prolongation, or fatal arrhythmia or autonomic impairment, increased thrombogenesis, inflammation, endothelial dysfunction, and vasoconstriction caused by aberrant endocrine or nervous reactions during recognised or unrecognised hypoglycaemic episodes (11, 102–113). All of these alterations can affect heart anatomy, cardiac stress, vascular hemodynamics, and function (13, 114–118). Episodes of asymptomatic or unreported symptomatic hypoglycaemia probably occur after the initial occurrence of SH, increasing the likelihood of recurrent incidents. Although these effects are true, they are more likely to explain a temporary increase in CV risk at the acute phase of hypoglycaemia rather than the long-term connection observed in another study (63).

Secondly, hypoglycaemia or SH is a plausible indicator instead of a direct cause of an elevated risk of unfavorable clinical outcomes, which reflects the influence of concomitant illnesses in addition to unmeasured or incompletely defined confounding variables (119) that independently increase the risk of mortality as opposed to being a risk factor causing these events. Other studies suggest that SH is only associated with cardiovascular events in individuals who already have a high cardiovascular risk (120, 121). Nonetheless, our subgroup analyses reveal a quantitative insignificant difference in the RR of all-cause mortality for those with low versus high cardiovascular risk (RR 1.62 vs. 1.94, respectively). This result suggests the effect may be more pronounced in in those with a greater cardiovascular risk.

The mechanisms that relate SH to non-cardiovascular fatalities remain unknown (22, 54). SH may be a signal for greater provider monitoring in clinical settings, or a sign of quickly deteriorating health. SH may be appropriate for clinicians to review the mental and physical states of a patient to see whether any therapy adjustment is required. Such actions may minimize the risks of future hypoglycaemia and CVDs.

Subgroup analyses were performed so as to clarify whether the incidence of cardiac arrhythmia or all-cause death differed among relevant characteristics. In the trials with T2DM patients, preliminary subgroup analysis revealed a substantial increase in overall death risk among hypoglycemic versus euglycaemic individuals. Because T1DM patients are frequently younger than T2DM patients, they have less risk factors for death. Despite this, SH is more likely to attack T1DM patients, raising the risk of arrhythmia. SH is more likely to assault T1DM patients, increasing the risk of arrhythmia. This is not the case, and the underlying reason is unknown (87). In terms of hypoglycemia episodes and severity, more episodes and severity lead to higher risks of arrhythmia and all-cause mortality rate, and may indicate quickly failing health. Shorter follow-up period is associated with a greater increase in all-cause mortality in hypoglycemic patients compared euglycaemic patients. It might be that the population with the shortest follow-up duration has the most co-morbidities or frailty, in whom severe hypoglycemia episodes may be a surrogate for the underlying severity of the overall advanced and complex illness condition. Subgroup analysis based on study design methodologies for extracting effect estimates calls for more well-designed research in the future.

No review or meta-analysis can avoid heterogeneity of OS due to the lack of standardisation of method for clarifying cases, study design and time, classification of endpoints, and the amount of inter-study confounding that has been adjusted. For example, definitions of hypoglycaemia differ greatly. In spite of the sensitivity testing, we were unable to account for the greatest between-study heterogeneity in the outcome of cardiac arrhythmia and death. Therefore, these results need confirmation from more research. Furthermore, the statistical methodologies used in p-h/sa of RCTs and OSs did not adequately address the influence of unmeasured confounding components regarding overall effect estimation. The origins of variability were attributed to differences in study populations and exposure, according to sensitivity analyses.

Some of the significant strengths of this meta-analysis include the detailed retrieval plan with Cochrane procedures, the broad search approach, the relatively large sample size, and the capacity to investigate the link between hypoglycemia and cardiac arrhythmia or death. Nevertheless, this study has some limitations. First, the avoidance of unreported reports can have influenced our findings. Second, there is also a wide range of exposure, including severe hypoglycemia and any hypoglycemia. Third, the analyses employed for arrhythmia source research have drawbacks as well. Some studies have presented incident rate ratios for arrhythmias, implying that an increased risk applies to the whole research group whereas, in fact, only a few individuals who are extremely vulnerable may be impacted (45). Fourth, because we did not test autonomic function, we were unable to investigate its potential contributions.

In summary, hypoglycemia, as opposed to euglycaemia, is associated with a higher risk of cardiac arrhythmia and mortality. Nevertheless, evidence of potential causal linkages beyond statistical associations must await proof by additional specifically well planned research that controls for all potential remaining confounding factors, including a unified definition of hypoglycemia (122) as recommended by the International Hypoglycaemia Study Group.

## Data availability statement

The raw data supporting the conclusions of this article will be made available by the authors, without undue reservation.

## Author contributions

XW and GL conceived the conception and design of the study, the search of the relevant literature, the extraction and analysis of data, and the drafting and revision of the final manuscript. SZ and FZ interpreted the analysed data, reviewed the manuscript critically, and contributed to its drafting. The final manuscript was read and approved by all authors.
