# Supplementary material for: Association of hypoglycaemia with the risks of arrhythmia and mortality in individuals with diabetes - a systematic review and meta-analysis
Source: Front Endocrinol (Lausanne). 2023 Aug 14;14:1222409. doi: 10.3389/fendo.2023.1222409 (PMC10461564; doi:10.3389/fendo.2023.1222409)
Supplement: Supplementary file 1 [file Table_1.doc]

Table S1 Quality assessment of studies using Newcastle-Ottawa Scale for Cohort Studies for Arrhythmias

| NOS scale | Chow et al(2014) | Tsujimoto et al(2015) | Amione et al(2017) | Ko et al(2018) | Novodvorsky et al(2017) | Lee et al(2018) | Zhang et al(2020) | Echouffo-Tcheugui et al(2021) | Abdelhamid et al(2021) |
| --- | --- | --- | --- | --- | --- | --- | --- | --- | --- |
| A-Selection (maximum 4*) |  |  |  |  |  |  |  |  |  |
| 1.Representativeness of general community population | 0 | * | * | * | 0 | * | * | * | 0 |
| 2.The reference group was drawn from the same community | * | * | * | * | * | * | * | * | * |
| 3.Ascertainment the exposure of hypoglycemia | * | * | * | * | * | * | * | * | * |
| 4.Outcome of interest was not present at baseline | * | * | * | * | * | * | * | * | * |
| B-Comparability (maximum 2*) |  |  |  |  |  |  |  |  |  |
| 5.Controlled for one variable | 0 | * | * | * | 0 | * | * | * | 0 |
| 6.Controlled for 2 or more variables | 0 | * | * | * | 0 | * | * | * | 0 |
| C-Outcome (maximum 3*) |  |  |  |  |  |  |  |  |  |
| 7. Outcome of interest was certificated by hospital or local municipal registration | * | * | * | * | * | * | * | * | * |
| 8. Adequate duration of follow-up(one year) | 0 | * | * | * | 0 | * | * | * | 0 |
| 9.Adequacy of follow-up rate (>90%) of cohorts | * | * | * | * | * | * | * | * | * |
| Total scores (maximum 9*) | 5 | 9 | 9 | 9 | 5 | 9 | 9 | 9 | 5 |

Notes: Newcastle-Ottawa Scale, NOS; “*” meant the study was corresponded to the NOS criteria,” 0” meant the study wasn’t correspond to the NOS criteria

Table S1 Quality assessment of studies using Newcastle-Ottawa Scale for Cohort Studies for Arrhythmias

| NOS scale | Andersen et al(2021) | Mylona et al(2020) |  |  |  |  |  |  |  |
| --- | --- | --- | --- | --- | --- | --- | --- | --- | --- |
| A-Selection (maximum 4*) |  |  |  |  |  |  |  |  |  |
| 1.Representativeness of general  community population | 0 | * |  |  |  |  |  |  |  |
| 2.The reference group was drawn  from the same community | * | * |  |  |  |  |  |  |  |
| 3.Ascertainment the exposure of hypoglycemia | * | * |  |  |  |  |  |  |  |
| 4.Outcome of interest was not present at baseline | * | * |  |  |  |  |  |  |  |
| B-Comparability (maximum 2*) |  |  |  |  |  |  |  |  |  |
| 5.Controlled for one variable | 0 | 0 |  |  |  |  |  |  |  |
| 6.Controlled for 2 or more variables | 0 | 0 |  |  |  |  |  |  |  |
| C-Outcome (maximum 3*) |  |  |  |  |  |  |  |  |  |
| 7. Outcome of interest was certificated by hospital or local municipal registration | * | * |  |  |  |  |  |  |  |
| 8. Adequate duration of follow-up(one year) | * | * |  |  |  |  |  |  |  |
| 9.Adequacy of follow-up rate (>90%) of cohorts | * | * |  |  |  |  |  |  |  |
| Total scores (maximum 9*) | 6 | 7 |  |  |  |  |  |  |  |

Notes: Newcastle-Ottawa Scale, NOS; “*” meant the study was corresponded to the NOS criteria,” 0” meant the study wasn’t correspond to the NOS criteria

Table S1 Quality assessment of studies using Newcastle-Ottawa Scale for Case-Control study for Arrhythmias

| NOS scale | Zhang et al(2020) |  |  |  |  |  |  |  |  |
| --- | --- | --- | --- | --- | --- | --- | --- | --- | --- |
| A-Selection (maximum 4*) |  |  |  |  |  |  |  |  |  |
| 1.The case definition was adequate | * |  |  |  |  |  |  |  |  |
| 2.The cases were consecutive or are obviously representative series of cases | * |  |  |  |  |  |  |  |  |
| 3.The reference group was drawn from the same community | * |  |  |  |  |  |  |  |  |
| 4. No history of endpoints were present in the reference group | * |  |  |  |  |  |  |  |  |
| B-Comparability (maximum 2*) |  |  |  |  |  |  |  |  |  |
| 5.Controlled for one variable | * |  |  |  |  |  |  |  |  |
| 6.Controlled for 2 or more variables | * |  |  |  |  |  |  |  |  |
| C-Exposure (maximum 3*) |  |  |  |  |  |  |  |  |  |
| 7. Exposure was certificated by hospital or local municipal registration | * |  |  |  |  |  |  |  |  |
| 8. Same method of ascertainment for cases and controls | * |  |  |  |  |  |  |  |  |
| 9. Same non-response rate for both groups | * |  |  |  |  |  |  |  |  |
| Total scores (maximum 9*) | 9 |  |  |  |  |  |  |  |  |

Notes: Newcastle-Ottawa Scale, NOS; “*” meant the study was corresponded to the NOS criteria,” 0” meant the study wasn’t correspond to the NOS criteria

Table S1 Quality assessment of studies using Newcastle-Ottawa Scale for Subanalyses or Post-hoc Analyses of RCTs for Arrhythmias

| NOS scale | Kaze et al(2022) |  |  |  |  |  |  |  |  |
| --- | --- | --- | --- | --- | --- | --- | --- | --- | --- |
| A-Selection (maximum 4*) |  |  |  |  |  |  |  |  |  |
| 1.Representativeness of general community population | * | * | * |  |  |  |  |  |  |
| 2.The reference group was drawn from the same community | * | * | * |  |  |  |  |  |  |
| 3.Ascertainment the exposure of hypoglycemia | * | * | * |  |  |  |  |  |  |
| 4.Outcome of interest was not present at baseline | * | * | * |  |  |  |  |  |  |
| B-Comparability (maximum 2*) |  |  |  |  |  |  |  |  |  |
| 5.Controlled for one variable | * | * | * |  |  |  |  |  |  |
| 6.Controlled for 2 or more variables | * | * | * |  |  |  |  |  |  |
| C-Outcome (maximum 3*) |  |  |  |  |  |  |  |  |  |
| 7. Outcome of interest was certificated by hospital or local municipal registration | * | * | * |  |  |  |  |  |  |
| 8. Adequate duration of follow-up(one year) | * | * | * |  |  |  |  |  |  |
| 9.Adequacy of follow-up rate (>90%) of cohorts | * | * | * |  |  |  |  |  |  |
| Total scores (maximum 9*) | 9 | 9 | 9 |  |  |  |  |  |  |

Notes: Newcastle-Ottawa Scale, NOS; “*” meant the study was corresponded to the NOS criteria,” 0” meant the study wasn’t correspond to the NOS criteria

Table S1 Quality assessment of studies using Agency for Healthcare Research and Quality for Cross-Sectional or Longitudinal Studies for Arrhythmias

| Item | GRUDEN et al(2012) | Pistrosch et al(2014) |  |  |  |  |
| --- | --- | --- | --- | --- | --- | --- |
| 1. Define the source of information (survey, record review) | Yes | Yes |  |  |  |  |
| 2. List the inclusion and exclusion criteria for exposed and unexposed subjects (cases and controls) or refer to previous publications. | Yes | Yes |  |  |  |  |
| 3. Indicate the period used for identifying the patients. | No | No |  |  |  |  |
| 4. Indicate whether or not the subjects were consecutive if not population-based. | Yes | Yes |  |  |  |  |
| 5. Indicate if the evaluators of the subjective components of study were masked to other aspects of the status of the participants. | Yes | Yes |  |  |  |  |
| 6. Describe any assessments undertaken for quality assurance purposes (e.g., test/retest of primary outcome measurements). | Yes | Yes |  |  |  |  |
| 7. Explain any patient exclusions from the analysis. | No | No |  |  |  |  |
| 8. Describe how confounding was assessed and/or controlled | No | No |  |  |  |  |
| 9. If applicable, explain how missing data were handled in the analysis. | No | No |  |  |  |  |
| 10. Summarize patient response rates and completeness of data collection. | Yes | Yes |  |  |  |  |
| 11. Clarify what follow-up, if any, was expected and the percentage of patients for which incomplete data or follow-up was obtained. | No | No |  |  |  |  |
| Total quality score/ Quality | 6 | 6 |  |  |  |  |

Notes: Y: Yes; N: No; UC: unclear

Table S1 Quality assessment of studies using Newcastle-Ottawa Scale for Cohort Studies for All-Cause Mortality

| NOS scale | Curkendall  et al(2009) | Li et al(2011) | Nirantharakumar  et al(2012) | ZHAO et al (2012) | Tan et al (2013) | HSU et al (2013) | Sechterberger et al (2013) | Lee et al (2014) | Kong et al (2014) | Lung et al (2014) |
| --- | --- | --- | --- | --- | --- | --- | --- | --- | --- | --- |
| A-Selection (maximum 4*) |  |  |  |  |  |  |  |  |  |  |
| 1.Representativeness of general  community population | * | 0 | 0 | * | 0 | * | 0 | * | * | * |
| 2.The reference group was drawn  from the same community | * | * | * | * | * | * | * | * | * | * |
| 3.Ascertainment the exposure of hypoglycemia | * | * | * | * | * | * | * | * | * | * |
| 4.Outcome of interest was not present at baseline | * | * | * | * | * | * | * | * | * | * |
| B-Comparability (maximum 2*) |  |  |  |  |  |  |  |  |  |  |
| 5.Controlled for one variable | * | 0 | 0 | * | 0 | 0 | * | * | * | 0 |
| 6.Controlled for 2 or more variables | * | 0 | 0 | * | 0 | 0 | * | * | * | 0 |
| C-Outcome (maximum 3*) |  |  |  |  |  |  |  |  |  |  |
| 7. Outcome of interest was certificated by hospital or local municipal registration | * | * | * | * | * | * | * | * | * | * |
| 8. Adequate duration of follow-up(one year) | * | 0 | 0 | * | * | * | 0 | * | * | * |
| 9.Adequacy of follow-up rate (>90%) of cohorts | * | * | * | * | * | * | * | * | * | * |
| Total scores (maximum 9*) | 9 | 5 | 5 | 9 | 5 | 7 | 7 | 9 | 9 | 7 |

Notes: Newcastle-Ottawa Scale, NOS; “*” meant the study was corresponded to the NOS criteria,” 0” meant the study wasn’t correspond to the NOS criteria

Table S1 Quality assessment of studies using Newcastle-Ottawa Scale for Cohort Studies for All-cause Mortality

| NOS scale | Cooper et al (2014) | Elwen et al (2015) | Khunti et al (2015) | Gómez-Huelgas et al (2015) | Escalada et al (2016) | Rauh et al (2016) | Takeishi et al (2016) | Cha et al (2016) | Sejling et al (2016) | Chevalier  et al (2016) |
| --- | --- | --- | --- | --- | --- | --- | --- | --- | --- | --- |
| A-Selection (maximum 4*) |  |  |  |  |  |  |  |  |  |  |
| 1.Representativeness of general  community population | * | 0 | * | * | * | * | 0 | * | * | * |
| 2.The reference group was drawn  from the same community | * | * | * | * | * | * | * | * | * | * |
| 3.Ascertainment the exposure of hypoglycemia | * | * | * | * | * | * | * | * | * | * |
| 4.Outcome of interest was not present at baseline | * | * | * | * | * | * | * | * | * | * |
| B-Comparability (maximum 2*) |  |  |  |  |  |  |  |  |  |  |
| 5.Controlled for one variable | * | 0 | * | 0 | 0 | * | * | * | 0 | * |
| 6.Controlled for 2 or more variables | * | 0 | * | 0 | 0 | * | * | * | 0 | * |
| C-Outcome (maximum 3*) |  |  |  |  |  |  |  |  |  |  |
| 7. Outcome of interest was certificated by hospital or local municipal registration | * | * | * | * | * | * | * | * | * | * |
| 8. Adequate duration of follow-up(one year) | * | * | * | 0 | * | * | * | * | * | * |
| 9.Adequacy of follow-up rate (>90%) of cohorts | * | * | * | * | * | * | * | * | * | * |
| Total scores (maximum 9*) | 9 | 6 | 9 | 6 | 7 | 9 | 8 | 9 | 7 | 9 |

Notes: Newcastle-Ottawa Scale, NOS; “*” meant the study was corresponded to the NOS criteria,” 0” meant the study wasn’t correspond to the NOS criteria

Table S1 Quality assessment of studies using Newcastle-Ottawa Scale for Cohort Studies for All-cause Mortality

| NOS scale | CHI et al (2017) | Leung et al (2019) | Lo et al (2019) | Mattishent et al (2019) | Wei et al (2019) | Yun et al (2019) | Wernly et al (2019) | Jensen et al (2020) | Zaccardi et al (2020) | Han et al (2022) |
| --- | --- | --- | --- | --- | --- | --- | --- | --- | --- | --- |
| A-Selection (maximum 4*) |  |  |  |  |  |  |  |  |  |  |
| 1.Representativeness of general  community population | * | 0 | * | * | 0 | * | 0 | * | * | * |
| 2.The reference group was drawn  from the same community | * | * | * | * | * | * | * | * | * | * |
| 3.Ascertainment the exposure of hypoglycemia | * | * | * | * | * | * | * | * | * | * |
| 4.Outcome of interest was not present at baseline | * | * | * | * | * | * | * | * | * | * |
| B-Comparability (maximum 2*) |  |  |  |  |  |  |  |  |  |  |
| 5.Controlled for one variable | * | 0 | * | * | * | * | 0 | * | 0 | * |
| 6.Controlled for 2 or more variables | * | 0 | * | * | * | * | 0 | * | 0 | * |
| C-Outcome (maximum 3*) |  |  |  |  |  |  |  |  |  |  |
| 7. Outcome of interest was certificated by hospital or local municipal registration | * | * | * | * | * | * | * | * | * | * |
| 8. Adequate duration of follow-up(one year) | * | * | * | * | * | * | * | * | * | * |
| 9.Adequacy of follow-up rate (>90%) of cohorts | * | * | * | * | * | * | * | * | * | * |
| Total scores (maximum 9*) | 9 | 6 | 9 | 9 | 8 | 9 | 6 | 9 | 7 | 9 |

Notes: Newcastle-Ottawa Scale, NOS; “*” meant the study was corresponded to the NOS criteria,” 0” meant the study wasn’t correspond to the NOS criteria

Table S1 Quality assessment of studies using Newcastle-Ottawa Scale for Cohort Studies for All-cause Mortality

| NOS scale | Cha et al (2022) |  |  |  |  |  |  |  |
| --- | --- | --- | --- | --- | --- | --- | --- | --- |
| A-Selection (maximum 4*) |  |  |  |  |  |  |  |  |
| 1.Representativeness of general  community population | * |  |  |  |  |  |  |  |
| 2.The reference group was drawn  from the same community | * |  |  |  |  |  |  |  |
| 3.Ascertainment the exposure of hypoglycemia | * |  |  |  |  |  |  |  |
| 4.Outcome of interest was not present at baseline | * |  |  |  |  |  |  |  |
| B-Comparability (maximum 2*) |  |  |  |  |  |  |  |  |
| 5.Controlled for one variable | 0 |  |  |  |  |  |  |  |
| 6.Controlled for 2 or more variables | 0 |  |  |  |  |  |  |  |
| C-Outcome (maximum 3*) |  |  |  |  |  |  |  |  |
| 7. Outcome of interest was certificated by hospital or local municipal registration | * |  |  |  |  |  |  |  |
| 8. Adequate duration of follow-up(one year) | * |  |  |  |  |  |  |  |
| 9.Adequacy of follow-up rate (>90%) of cohorts | * |  |  |  |  |  |  |  |
| Total scores (maximum 9*) | 7 |  |  |  |  |  |  |  |

Notes: Newcastle-Ottawa Scale, NOS; “*” meant the study was corresponded to the NOS criteria,” 0” meant the study wasn’t correspond to the NOS criteria

Table S1 Quality assessment of studies using Newcastle-Ottawa Scale for Case-Control study for All-cause Mortality

| NOS scale | MCCOY  et al (2012) | Lu et al (2016) | Ferreira et al (2019) |  |  |  |  |  |  |
| --- | --- | --- | --- | --- | --- | --- | --- | --- | --- |
| A-Selection (maximum 4*) |  |  |  |  |  |  |  |  |  |
| 1.The case definition was adequate | * | * | 0 |  |  |  |  |  |  |
| 2.The cases were consecutive or are obviously representative series of cases | * | * | * |  |  |  |  |  |  |
| 3.The reference group was drawn from the same community | * | * | * |  |  |  |  |  |  |
| 4. No history of endpoints were present in the reference group | * | * | * |  |  |  |  |  |  |
| B-Comparability (maximum 2*) |  |  |  |  |  |  |  |  |  |
| 5.Controlled for age | * | * | 0 |  |  |  |  |  |  |
| 6.Controlled for 2 or more variables | * | * | 0 |  |  |  |  |  |  |
| C-Exposure (maximum 3*) |  |  |  |  |  |  |  |  |  |
| 7. Exposure was certificated by hospital or local municipal registration | * | * | * |  |  |  |  |  |  |
| 8. Same method of ascertainment for cases and controls | * | * | * |  |  |  |  |  |  |
| 9. Same non-response rate for both groups | * | * | * |  |  |  |  |  |  |
| Total scores (maximum 9*) | 9 | 9 | 6 |  |  |  |  |  |  |

Notes: Newcastle-Ottawa Scale, NOS; “*” meant the study was corresponded to the NOS criteria,” 0” meant the study wasn’t correspond to the NOS criteria

Table S1 Quality assessment of studies using Newcastle-Ottawa Scale for subanalyses or post-hoc analyses of RCTs for All-cause Mortality

| NOS scale | Mellbin et al(2009) | Bonds et al(2010) | Zoungas  et al(2010) | ORIGIN Trial Investigators  et al (2013) | Standl et al (2018) | Zinman et al (2018) | Pieber et al (2018) | Davis et al (2019) | Standl et al (2020) | Heller et al (2022) |
| --- | --- | --- | --- | --- | --- | --- | --- | --- | --- | --- |
| A-Selection (maximum 4*) |  |  |  |  |  |  |  |  |  |  |
| 1.Representativeness of general  community population | * | * | * | * | * | * | * | * | * | * |
| 2.The reference group was drawn  from the same community | * | * | * | * | * | * | * | * | * | * |
| 3.Ascertainment the exposure of hypoglycemia | * | * | * | * | * | * | * | * | * | * |
| 4.Outcome of interest was not present at baseline | * | * | * | * | * | * | * | * | * | * |
| B-Comparability (maximum 2*) |  |  |  |  |  |  |  |  |  |  |
| 5.Controlled for one variable | * | * | * | * | * | * | 0 | * | * | 0 |
| 6.Controlled for 2 or more variables | * | * | * | * | * | * | 0 | * | * | 0 |
| C-Outcome (maximum 3*) |  |  |  |  |  |  |  |  |  |  |
| 7. Outcome of interest was certificated by hospital or local municipal registration | * | * | * | * | * | * | * | * | * | * |
| 8. Adequate duration of follow-up(one year) | * | * | * | * | * | * | * | * | * | * |
| 9.Adequacy of follow-up rate (>90%) of cohorts | * | * | * | * | * | * | * | 0 | * | * |
| Total scores (maximum 9*) | 9 | 9 | 9 | 9 | 9 | 9 | 7 | 8 | 9 | 7 |

Notes: Newcastle-Ottawa Scale, NOS; “*” meant the study was corresponded to the NOS criteria,” 0” meant the study wasn’t correspond to the NOS criteria

Table S1 Quality assessment of studies using Agency for Healthcare Research and Quality for Cross-Sectional or Longitudinal Studies for All-cause Mortality

| Item | Freemantle et al (2016) | Zhao et al (2017) |  |  |  |
| --- | --- | --- | --- | --- | --- |
| 1. Define the source of information (survey, record review) | Yes | Yes |  |  |  |
| 2. List the inclusion and exclusion criteria for exposed and unexposed subjects (cases and controls) or refer to previous publications. | Yes | Yes |  |  |  |
| 3. Indicate the period used for identifying the patients. | No | No |  |  |  |
| 4. Indicate whether or not the subjects were consecutive if not population-based. | Yes | Yes |  |  |  |
| 5. Indicate if the evaluators of the subjective components of study were masked to other aspects of the status of the participants. | Yes | Yes |  |  |  |
| 6. Describe any assessments undertaken for quality assurance purposes (e.g., test/retest of primary outcome measurements). | Yes | Yes |  |  |  |
| 7. Explain any patient exclusions from the analysis. | No | No |  |  |  |
| 8. Describe how confounding was assessed and/or controlled | No | No |  |  |  |
| 9. If applicable, explain how missing data were handled in the analysis. | No | No |  |  |  |
| 10. Summarize patient response rates and completeness of data collection. | Yes | Yes |  |  |  |
| 11. Clarify what follow-up, if any, was expected and the percentage of patients for which incomplete data or follow-up was obtained. | No | No |  |  |  |
| Total quality score/ Quality | 6 | 6 |  |  |  |

Notes: Y: Yes; N: No; UC: unclear
