# Supplementary material for: Association of hypoglycaemia with the risks of arrhythmia and mortality in individuals with diabetes - a systematic review and meta-analysis
Source: Front Endocrinol (Lausanne). 2023 Aug 14;14:1222409. doi: 10.3389/fendo.2023.1222409 (PMC10461564; doi:10.3389/fendo.2023.1222409)
Supplement: Supplementary file 2 [file DataSheet_1.docx]

**Search strategy**

**Pubmed**

#1 “Hypoglycemia”[MeSH Terms]

#2 hypoglycemia[Title/Abstract] OR hypoglycaemia[Title/Abstract] OR hypogly*[Title/Abstract] OR hyperinsulin*[Title/Abstract] OR hyper-insulin*[Title/Abstract] OR "insulin coma*"[Title/Abstract] OR nesidioblastos*[Title/Abstract] OR "low blood sugar"[Title/Abstract] OR "low blood glucose"[Title/Abstract] OR "Glyc* Variability"[Title/Abstract] OR "Glucose Variability"[Title/Abstract]

#3 #1 OR #2

#4 NIDDM[Title/Abstract] OR MODY[Title/Abstract] OR “late onset diabet*”[Title/Abstract] OR “maturity onset diabet*”[Title/Abstract] OR “non insulin* depend*”[Title/Abstract] OR “noninsulin* depend*”[Title/Abstract] OR " type 2 diabet*”[Title/Abstract] OR “type II diabet*”[Title/Abstract] OR T2D[Title/Abstract] OR T2DM[Title/Abstract] OR diabetic[Title/Abstract] OR “insulin resistance”[Title/Abstract]
#5 "Diabetes mellitus, type 2"[MeSH Terms]

#6 "glucose intolerance"[MeSH Terms]
#7 “insulin resistance”[MeSH Terms]

#8 #4 OR #5 OR #6 OR #7

#9 "Diabetes mellitus, type 1"[MeSH Terms]

#10 "Diabetic Ketoacidosis"[MeSH Terms]

#11 "Diabetes Complications"[MeSH Terms]

#12 IDDM[Title/Abstract] OR T1DM[Title/Abstract] OR T1D[Title/Abstract] OR “typ* 1 diabet*”[Title/Abstract] OR “typ* I diabet*”[Title/Abstract] OR “insulin* depend*”[Title/Abstract] OR “insulin?depend*”[Title/Abstract] OR “earl* diabet*”[Title/Abstract] OR “auto?immun* diabet*”[Title/Abstract] OR “sudden onset diabet*”[Title/Abstract] OR “insulin* defic* absolut*”[Title/Abstract] OR “acidos* diabet”[Title/Abstract] OR “juvenil* diabet*”[Title/Abstract] OR “child* diabet*”[Title/Abstract] OR “keto* diabet*”[Title/Abstract] OR “labil* diabet”*[Title/Abstract] OR “britt* diabet*”[Title/Abstract]

#13 #9 OR #10 OR #11 OR #12

#14 #8 OR #13

#15 "Arrhythmias, Cardiac"[MeSH Terms]

#16 "Atrial Flutter"[MeSH Terms]

#17 "Tachycardia, Ectopic Atrial"[MeSH Terms]

#18 "Atrial Fibrillation"[MeSH Terms] OR "Ventricular Fibrillation"[MeSH Terms]

#19 "Tachycardia, Supraventricular"[MeSH Terms]

#20 "Cardiac Complexes, Premature"[MeSH Terms]

#21 "Ventricular Premature Complexes"[MeSH Terms]

#22 "Atrial Premature Complexes"[MeSH Terms]

#23 "Ventricular Dysfunction, Left"[MeSH Terms]

#24 "Bradycardia"[MeSH Terms]

#25 "Heart Block"[MeSH Terms]

#26 "Arrhythmia, Sinus"[MeSH Terms]

#27 "Pre-Excitation Syndromes"[MeSH Terms]

#28 "Long QT syndrome"[MeSH Terms]

#29 "Sick Sinus Syndrome"[MeSH Terms]

#30 "Brugada Syndrome"[MeSH Terms]

#31 "Heart Arrhythmia*"[Title/Abstract] OR "Heart Dysrhythmia*"[Title/Abstract] OR Arrhythmia*[Title/Abstract] OR tachycardia*[Title/Abstract] OR tachyarrhythmia*[Title/Abstract] OR Dysrhythmia*[Title/Abstract]

#32 "atrial fibrillation"[Title/Abstract] OR "Ventricular fibrillation"[Title/Abstract] OR "atrial flutter*"[Title/Abstract] OR "atrial tachycardia*"[Title/Abstract] OR "supraventricular tachycardia*"[Title/Abstract] OR "supraventricular tachyarrhythmia*"[Title/Abstract] OR "atrial tachyarrhythmia*"[Title/Abstract] OR "Ventricular tachycardia*"[Title/Abstract] OR "Ventricular tachyarrhythmia*"[Title/Abstract] OR AF[Title/Abstract] OR "atrium fibrillation"[Title/Abstract] OR "atrium flutter*"[Title/Abstract] OR "atrium tachycardia*"[Title/Abstract] OR "atrium tachyarrhythmia*"[Title/Abstract] OR "auricular fibrillation"[Title/Abstract] OR "auricular flutter*"[Title/Abstract] OR "auricular tachycardia*"[Title/Abstract] OR "auricular tachyarrhythmia*"[Title/Abstract]

#33 "Ventricular Premature Beat*"[Title/Abstract] OR VPB*[Title/Abstract] OR "ventricular premature contraction*"[Title/Abstract] OR "Premature Ventricular Complex*"[Title/Abstract] OR "Premature Ventricular Contraction*"[Title/Abstract] OR "Atrial Premature Beat*"[Title/Abstract] OR "Atrial Premature Contraction*"[Title/Abstract] OR "Atrial Premature Complex*"[Title/Abstract] OR "Premature Atrial Contraction*"[Title/Abstract] OR "Abnormal Heart Rhythm*"[Title/Abstract] OR Arrhythmogenesis[Title/Abstract] OR "Atrial Ectopic Beat*"[Title/Abstract] OR Bradycardia[Title/Abstract]

#34 "Cardiac Rhythm Disturbance*"[Title/Abstract] OR "Heart Rate Variability"[Title/Abstract] OR HRV[Title/Abstract] OR "Heart Block"[Title/Abstract] OR "Irregular Heartbeat*"[Title/Abstract] OR "Long QT Syndrome"[Title/Abstract] OR "Corrected QT Interval"[Title/Abstract] OR "QT Interval"[Title/Abstract] OR "QT Interval Prolongation"[Title/Abstract] OR QTc[Title/Abstract]

#35 "ECG Abnormalit*"[Title/Abstract] OR "Electrocardiogr* Abnormalit*"[Title/Abstract] OR "ECG Disturbance*"[Title/Abstract] OR "Electrocardiogr* Disturbance*"[Title/Abstract] OR "ECG Alteration*"[Title/Abstract] OR "Electrocardiogr* Alteration*"[Title/Abstract] OR "Electrocardiogr* Change*"[Title/Abstract] OR "ECG Change*"[Title/Abstract]

#36 "Left Ventricular Dysfunction"[Title/Abstract] OR Proarrhythmi*[Title/Abstract] OR Pro-arrhythmi*[Title/Abstract] OR "Ventricular Electrophysiological Disturbance*"[Title/Abstract] OR "Ventricular Extrasystole*"[Title/Abstract] OR "Heart Rate Abnormali*"[Title/Abstract] OR "Cardiac Autonomic Dysfunction"[Title/Abstract] OR "Cradiac Dysfunction"[Title/Abstract] OR "Heart Dysfunction"[Title/Abstract] OR "Cardiac Abnormalit*"[Title/Abstract] OR "Heart Abnormalit*"[Title/Abstract] OR "Heart Autonomic Dysfunction"[Title/Abstract]

#37 "Sinus Arrhythmia*"[Title/Abstract] OR "Pre_Excitation Syndrome"[Title/Abstract] OR "Cardiac Reploari*ation"[Title/Abstract] OR "Heart Repolari*ation"[Title/Abstract] OR "Sick Sinus Syndrome"[Title/Abstract] OR "Brugada Syndrome"[Title/Abstract] OR "Premature cardiac Complex"[Title/Abstract] OR "Bundle Branch Block"[Title/Abstract] OR "Atrioventricular block"[Title/Abstract] OR "AV Block"[Title/Abstract] OR "Sinoatrial Block"[Title/Abstract] OR "Cardiac Complex* Premature"[Title/Abstract] OR Parasystole[Title/Abstract] OR "Commotio Cordis"[Title/Abstract]

#38 #15 OR #16 OR #17 OR #18 OR #19 OR #20 OR #21 OR #22 OR #23 OR #24 OR #25 OR #26 OR #27 OR #28 OR #29 OR #30 OR #31 OR #32 OR #33 OR #34 OR #35 OR #36 OR #37

#39 “death”[Title/Abstract] OR “decease”[Title/Abstract] OR “demise”[Title/Abstract] OR “die”[Title/Abstract] OR “dead”[Title/Abstract] OR “loss of life”[Title/Abstract] OR “life time”[Title/Abstract] OR “lifetime”[Title/Abstract] OR “life span”[Title/Abstract] OR “duration of life”[Title/Abstract] OR “mortality”[Title/Abstract] OR “mortalit*”[Title/Abstract] OR “length of life”[Title/Abstract] OR “life duration” [Title/Abstract] OR “longevity”[Title/Abstract] OR “natural life”[Title/Abstract] OR “operating life”[Title/Abstract] OR “survival”[Title/Abstract]

#40 “death”[MeSH Terms]

#41 "[Mortality](https://www.ncbi.nlm.nih.gov/mesh/68009026)"[MeSH Terms]

#42 #39 OR #40 OR #41

#43 #38 OR #42

#44 (animal[mh] not human[mh])

#45 comment[Publication Type] OR editorial[Publication Type] OR letter[Publication Type]

#46 #3 AND #14 AND #43 NOT#44 NOT #45

**Embase**

#1 'Hypoglycemia’/exp

#2 Hypoglycemia:ab,ti OR Hypoglycaemia:ab,ti OR hypogly$:ab,ti OR hyperinsulin$:ab,ti OR hyper-insulin*:ab,ti OR 'insulin coma$':ab,ti OR nesidioblastos$:ab,ti OR 'low blood sugar':ab,ti OR 'low blood glucose':ab,ti OR 'Glyc* Variability':ab,ti OR 'Glucose Variability':ab,ti

#3 #1 OR #2

#4 NIDDM:ab,ti OR MODY:ab,ti OR “late onset diabet$”:ab,ti OR “maturity onset diabet$”:ab,ti OR “non insulin$ depend$”:ab,ti OR “noninsulin$ depend$”:ab,ti OR " type 2 diabet$”:ab,ti OR “type II diabet$”:ab,ti OR T2D:ab,ti OR T2DM:ab,ti OR diabetic:ab,ti OR “insulin resistance”:ab,ti
#5 ‘non insulin dependent diabetes mellitus’/exp

#6 ‘glucose intolerance’/exp
#7 ‘insulin resistance’/exp

#8 #4 OR #5 OR #6 OR #7

#9 ‘insulin dependent diabetes mellitus’/exp

#10 ‘Diabetic Ketoacidosis’/exp

#11 ‘diabetic complication’/exp

#12 IDDM:ab,ti OR T1DM:ab,ti OR T1D:ab,ti OR “typ$ 1 diabet$”:ab,ti OR 'typ$ I diabet$':ab,ti OR 'insulin$ depend$':ab,ti OR 'insulin?depend$':ab,ti OR 'earl$ diabet$':ab,ti OR 'auto?immun$ diabet$':ab,ti OR 'sudden onset diabet$':ab,ti OR 'insulin$ defic$ absolut$':ab,ti OR 'acidos$ diabet':ab,ti OR 'juvenil$ diabet$':ab,ti OR 'child$ diabet$':ab,ti OR 'keto$ diabet$':ab,ti OR 'labil$ diabet$':ab,ti OR 'britt$ diabet$':ab,ti

#13 #9 OR #10 OR #11 OR #12

#14 #8 OR #13

#15 'heart arrhythmia’/exp

#16 'heart atrium flutter'/exp

#17 'ectopic atrial tachycardia’/exp

### #18 'Atrial Fibrillation'/exp

### #19 '[heart ventricle fibrillation](http://www.baidu.com/link?url=2y6mPFAkttS_L7BXGud96WQhgwGatWJkGHbY1iRfvQ0HSHfk1VPtGOCjj1KqB6pclqkM720uFjQJfERveI2e3Xg9KVCxnGal8MYnBzfnmdUOHTPwyo__cEKOdF5VbnFi)'/exp

#20 'supraventricular tachycardia'/exp

#21 'supraventricular premature beat'/exp

#22 'heart ventricle extrasystole'/exp

#23 'heart left ventricle failure'/exp

#24 'Bradycardia'/exp

#25 'Heart block'/exp

#26 'sinus arrhythmia'/exp

#27 'heart preexcitation'/exp

#28 'long QT syndrome'/exp

#29 'sick sinus syndrome'/exp

#30 'Brugada syndrome'/exp

#31 'Heart Arrhythmia$':ab,ti OR 'Heart Dysrhythmia$':ab,ti OR Arrhythmia$:ab,ti OR tachycardia$:ab,ti OR tachyarrhythmia$:ab,ti OR Dysrhythmia$:ab,ti

#32 'atrial fibrillation':ab,ti OR 'Ventricular fibrillation':ab,ti OR 'atrial flutter$':ab,ti OR 'atrial tachycardia$':ab,ti OR 'supraventricular tachycardia$':ab,ti OR 'supraventricular tachyarrhythmia$':ab,ti OR 'atrial tachyarrhythmia$':ab,ti OR 'Ventricular tachycardia$':ab,ti OR 'Ventricular tachyarrhythmia$':ab,ti OR AF:ab,ti OR 'atrium fibrillation':ab,ti OR 'atrium flutter$':ab,ti OR 'atrium tachycardia$':ab,ti OR 'atrium tachyarrhythmia$':ab,ti OR 'auricular fibrillation':ab,ti OR 'auricular flutter$':ab,ti OR 'auricular tachycardia$':ab,ti OR 'auricular tachyarrhythmia$':ab,ti

#33 'Ventricular Premature Beat$':ab,ti OR VPB$:ab,ti OR 'ventricular premature contraction$':ab,ti OR 'Premature Ventricular Complex$':ab,ti OR 'Premature Ventricular Contraction$':ab,ti OR 'Atrial Premature Beat$':ab,ti OR 'Atrial Premature Contraction$':ab,ti OR 'Atrial Premature Complex$':ab,ti OR 'Premature Atrial Contraction$':ab,ti OR 'Abnormal Heart Rhythm$':ab,ti OR Arrhythmogenesis:ab,ti OR 'Atrial Ectopic Beat$':ab,ti OR Bradycardia:ab,ti

#34 'Cardiac Rhythm Disturbance$':ab,ti OR 'Heart Rate Variability':ab,ti OR HRV:ab,ti OR 'Heart Block':ab,ti OR 'Irregular Heartbeat$':ab,ti OR 'Long QT Syndrome':ab,ti OR 'Corrected QT Interval':ab,ti OR 'QT Interval':ab,ti OR 'QT Interval Prolongation':ab,ti OR QTc:ab,ti

#35 'ECG Abnormalit$':ab,ti OR 'Electrocardiogr$ Abnormalit$':ab,ti OR 'ECG Disturbance$':ab,ti OR 'Electrocardiogr$ Disturbance$':ab,ti OR 'ECG Alteration$':ab,ti OR 'Electrocardiogr$ Alteration$':ab,ti OR 'Electrocardiogr$ Change$':ab,ti OR 'ECG Change$':ab,ti

#36 'Left Ventricular Dysfunction':ab,ti OR Proarrhythmi$:ab,ti OR Pro-arrhythmi*:ab,ti OR 'Ventricular Electrophysiological Disturbance$':ab,ti OR 'Ventricular Extrasystole$':ab,ti OR 'Heart Rate Abnormali$':ab,ti OR 'Cardiac Autonomic Dysfunction':ab,ti OR 'Cradiac Dysfunction':ab,ti OR 'Heart Dysfunction':ab,ti OR 'Cardiac Abnormalit$':ab,ti OR 'Heart Abnormalit$':ab,ti OR 'Heart Autonomic Dysfunction':ab,ti

#37 'Sinus Arrhythmia$':ab,ti OR 'Pre_Excitation Syndrome':ab,ti OR 'Cardiac Reploari$ation':ab,ti OR 'Heart Repolari$ation':ab,ti OR 'Sick Sinus Syndrome':ab,ti OR 'Brugada Syndrome':ab,ti OR 'Premature cardiac Complex':ab,ti OR 'Bundle Branch Block':ab,ti OR 'Atrioventricular block':ab,ti OR 'AV Block':ab,ti OR 'Sinoatrial Block':ab,ti OR 'Cardiac Complex$ Premature':ab,ti OR Parasystole:ab,ti OR 'Commotio Cordis':ab,ti

#38 #15 OR #16 OR #17 OR #18 OR #19 OR #20 OR #21 OR #22 OR #23 OR #24 OR #25 OR #26 OR #27 OR #28 OR #29 OR #30 OR #31 OR #32 OR #33 OR #34 OR #35 OR #36 OR #37

#39 'death':ab,ti OR 'decease':ab,ti OR 'demise':ab,ti OR 'die':ab,ti OR 'dead':ab,ti OR 'loss of life':ab,ti OR 'life time':ab,ti OR 'lifetime':ab,ti OR 'life span':ab,ti OR 'duration of life':ab,ti OR 'mortality':ab,ti OR 'mortalit$':ab,ti OR 'length of life':ab,ti OR 'life duration':ab,ti OR 'longevity':ab,ti OR 'natural life':ab,ti OR 'operating life':ab,ti OR 'survival':ab,ti

#40 ‘death’/exp OR ‘mortality’/exp

#41 #39 OR #40

#42 #38 OR #41

#43 #3 AND #14 AND #42

Lim #43 to human

**Cochrane**

#1 MeSH descriptor: [Hypoglycemia] explode all trees

#2 hypoglycemia:ti,ab,kw OR hypoglycaemia:ti,ab,kw OR hypogly*:ti,ab,kw OR hyperinsulin*:ti,ab,kw OR hyper-insulin*:ti,ab,kw OR (insulin coma*):ti,ab,kw OR nesidioblastos*:ti,ab,kw OR (low blood sugar):ti,ab,kw OR (low blood glucose):ti,ab,kw OR (Glyc* Variability):ti,ab,kw OR (Glucose Variability):ti,ab,kw

#3 #1 OR #2

#4 NIDDM:ti,ab,kw OR MODY:ti,ab,kw OR (late onset diabet*):ti,ab,kw OR (maturity onset diabet*):ti,ab,kw OR (non insulin* depend*):ti,ab,kw OR (noninsulin* depend*):ti,ab,kw OR (type 2 diabet*):ti,ab,kw OR (type II diabet*):ti,ab,kw OR T2D:ti,ab,kw OR T2DM:ti,ab,kw OR diabetic:ti,ab,kw OR (insulin resistance):ti,ab,kw
#5 MeSH descriptor:[Diabetes mellitus, type 2] explode all trees

#6 MeSH descriptor:[glucose intolerance] explode all trees
#7 MeSH descriptor:[insulin resistance] explode all trees

#8 #4 OR #5 OR #6 OR #7

#9 MeSH descriptor:[Diabetes mellitus, type 1] explode all trees

#10 MeSH descriptor: [Diabetic Ketoacidosis] explode all trees

#11 MeSH descriptor:[Diabetes Complications] explode all trees

#12 IDDM:ti,ab,kw OR T1DM:ti,ab,kw OR T1D:ti,ab,kw OR (typ* 1 diabet*):ti,ab,kw OR (typ* I diabet*):ti,ab,kw OR (insulin* depend*):ti,ab,kw OR (insulin?depend*):ti,ab,kw OR (earl* diabet*):ti,ab,kw OR (auto?immun* diabet*):ti,ab,kw OR (sudden onset diabet*):ti,ab,kw OR (insulin* defic* absolut*):ti,ab,kw OR (acidos* diabet):ti,ab,kw OR (juvenil* diabet*):ti,ab,kw OR (child* diabet*):ti,ab,kw OR (keto* diabet*):ti,ab,kw OR (labil* diabet)*:ti,ab,kw OR (britt* diabet*):ti,ab,kw

#13 #9 OR 10 OR #11 OR #12

#14 #8 OR #13

#15 MeSH descriptor: [Arrhythmias, Cardiac] explode all trees

#16 MeSH descriptor: [Atrial Flutter] explode all trees

#17 MeSH descriptor: [Tachycardia, Ectopic Atrial] explode all trees

#18 MeSH descriptor:[Atrial Fibrillation] explode all trees

#19 MeSH descriptor: [Ventricular Fibrillation] explode all trees

#20 MeSH descriptor: [Cardiac Complex, Premature] explode all trees

#21 MeSH descriptor: [Ventricular Premature Complexes] explode all trees

#22 MeSH descriptor: [Atrial Premature Complexes] explode all trees

#23 MeSH descriptor: [Ventricular Dysfunction, Left] explode all trees

#24 MeSH descriptor: [Bradycardia] explode all trees

#25 MeSH descriptor: [Heart Block] explode all trees

#26 MeSH descriptor: [Arrhythmia, Sinus] explode all trees

#27 MeSH descriptor: [Pre-Excitation Syndromes] explode all trees

#28 MeSH descriptor: [Long QT Syndrome] explode all trees

#29 MeSH descriptor: [Sick Sinus Syndrome] explode all trees

#30 MeSH descriptor: [Brugada Syndrome] explode all trees

#31 MeSH descriptor: [Tachycardia, Supraventricular] explode all trees

#32 (Heart Arrhythmia*):ti,ab,kw OR (Heart Dysrhythmia*):ti,ab,kw OR Arrhythmia*:ti,ab,kw OR tachycardia*:ti,ab,kw OR tachyarrhythmia*:ti,ab,kw OR Dysrhythmia*:ti,ab,kw

#33 (atrial fibrillation):ti,ab,kw OR (Ventricular fibrillation):ti,ab,kw OR (atrial flutter*):ti,ab,kw OR (atrial tachycardia*):ti,ab,kw OR (supraventricular tachycardia*):ti,ab,kw OR (supraventricular tachyarrhythmia*):ti,ab,kw OR (atrial tachyarrhythmia*):ti,ab,kw OR (Ventricular tachycardia*):ti,ab,kw OR (Ventricular tachyarrhythmia*):ti,ab,kw OR AF:ti,ab,kw OR (atrium fibrillation):ti,ab,kw OR (atrium flutter*):ti,ab,kw OR (atrium tachycardia*):ti,ab,kw OR (atrium tachyarrhythmia*):ti,ab,kw OR (auricular fibrillation):ti,ab,kw OR (auricular flutter*):ti,ab,kw OR (auricular tachycardia*):ti,ab,kw OR (auricular tachyarrhythmia*):ti,ab,kw

#34 (Ventricular Premature Beat*):ti,ab,kw OR VPB*:ti,ab,kw OR (ventricular premature contraction*):ti,ab,kw OR (Premature Ventricular Complex*):ti,ab,kw OR (Premature Ventricular Contraction*):ti,ab,kw OR (Atrial Premature Beat*):ti,ab,kw OR (Atrial Premature Contraction*):ti,ab,kw OR (Atrial Premature Complex*):ti,ab,kw OR (Premature Atrial Contraction*):ti,ab,kw OR (Abnormal Heart Rhythm*):ti,ab,kw OR Arrhythmogenesis:ti,ab,kw OR (Atrial Ectopic Beat*):ti,ab,kw OR Bradycardia:ti,ab,kw

#35 (Cardiac Rhythm Disturbance*):ti,ab,kw OR (Heart Rate Variability):ti,ab,kw OR HRV:ti,ab,kw OR (Heart Block):ti,ab,kw OR (Irregular Heartbeat*):ti,ab,kw OR (Long QT Syndrome):ti,ab,kw OR (Corrected QT Interval):ti,ab,kw OR (QT Interval):ti,ab,kw OR (QT Interval Prolongation):ti,ab,kw OR QTc:ti,ab,kw

#36 (ECG Abnormalit*):ti,ab,kw OR (Electrocardiogr* Abnormalit*):ti,ab,kw OR (ECG Disturbance*):ti,ab,kw OR (Electrocardiogr* Disturbance*):ti,ab,kw OR (ECG Alteration*):ti,ab,kw OR (Electrocardiogr* Alteration*):ti,ab,kw OR (Electrocardiogr* Change*):ti,ab,kw OR (ECG Change*):ti,ab,kw

#37 (Left Ventricular Dysfunction):ti,ab,kw OR Proarrhythmi*:ti,ab,kw OR Pro-arrhythmi*:ti,ab,kw OR (Ventricular Electrophysiological Disturbance*):ti,ab,kw OR (Ventricular Extrasystole*):ti,ab,kw OR (Heart Rate Abnormali*):ti,ab,kw OR (Cardiac Autonomic Dysfunction):ti,ab,kw OR (Cradiac Dysfunction):ti,ab,kw OR (Heart Dysfunction):ti,ab,kw OR (Cardiac Abnormalit*):ti,ab,kw OR (Heart Abnormalit*):ti,ab,kw OR (Heart Autonomic Dysfunction):ti,ab,kw

#38 (Sinus Arrhythmia*):ti,ab,kw OR (Pre_Excitation Syndrome):ti,ab,kw OR (Cardiac Reploari*ation):ti,ab,kw OR (Heart Repolari*ation):ti,ab,kw OR (Sick Sinus Syndrome):ti,ab,kw OR (Brugada Syndrome):ti,ab,kw OR (Premature cardiac Complex):ti,ab,kw OR (Bundle Branch Block):ti,ab,kw OR (Atrioventricular block):ti,ab,kw OR (AV Block):ti,ab,kw OR (Sinoatrial Block):ti,ab,kw OR (Cardiac Complex* Premature):ti,ab,kw OR Parasystole:ti,ab,kw OR (Commotio Cordis):ti,ab,kw

#39 #15 OR #16 OR #17 OR #18 OR #19 OR #20 OR #21 OR #22 OR #23 OR #24 OR #25 OR #26 OR #27 OR #28 OR #29 OR #30 OR #31 OR #32 OR #33 OR #34 OR #35 OR #36 OR #37 OR #38

#40 (death):ti,ab,kw OR (decease):ti,ab,kw OR (demise):ti,ab,kw OR (die):ti,ab,kw OR (dead):ti,ab,kw OR (loss of life):ti,ab,kw OR (life time):ti,ab,kw OR (lifetime):ti,ab,kw OR (life span):ti,ab,kw OR (duration of life):ti,ab,kw OR (mortality):ti,ab,kw OR (mortalit*):ti,ab,kw OR (length of life):ti,ab,kw OR (life duration):ti,ab,kw OR (longevity):ti,ab,kw OR (natural life):ti,ab,kw OR (operating life):ti,ab,kw OR (survival):ti,ab,kw

#41 MeSH descriptor:[death] explode all trees

#42 MeSH descriptor:[Mortality] explode all trees

#43 #40 OR #41 OR #42

#44 #39 OR #43

#45 #3 AND #14 AND #44

#46 Limit #45 to clinical trails

**SCOPUS**

TITLE-ABS-KEY(hypoglycemia OR hypoglycaemia OR hypogly* OR hyperinsulin* OR hyper-insulin* OR "insulin coma*" OR nesidioblastos* OR "low blood sugar" OR "low blood glucose" OR "Glyc* Variability" OR "Glucose Variability") AND (TITLE-ABS-KEY(NIDDM OR MODY OR “glucose intolerance” OR “late onset diabet*” OR “maturity onset diabet*” OR “non insulin* depend*” OR “noninsulin* depend*” OR “type 2 diabet*” OR “type II diabet*” OR “insulin resistance”) OR TITLE-ABS-KEY(IDDM OR T1DM OR T1D OR “typ* 1 diabet*” OR “typ* I diabet*” OR “insulin* depend*” OR “insulin?depend*” OR “earl* diabet*” OR “auto?immun* diabet*” OR “sudden onset diabet*” OR “insulin* defic* absolut*” OR “acidos* diabet” OR “juvenil* diabet*” OR “child* diabet*” OR “keto* diabet*” OR “labil* diabet*” OR “britt* diabet*”)) AND (TITLE-ABS-KEY("Heart Arrhythmia*" OR "Heart Dysrhythmia*" OR Arrhythmia* OR tachycardia* OR tachyarrhythmia* OR Dysrhythmia* OR "atrial fibrillation" OR "Ventricular fibrillation" OR "atrial flutter*" OR "atrial tachycardia*" OR "supraventricular tachycardia*" OR "supraventricular tachyarrhythmia*" OR "atrial tachyarrhythmia*" OR "Ventricular tachycardia*" OR "Ventricular tachyarrhythmia*" OR AF OR "atrium fibrillation" OR "atrium flutter*" OR "atrium tachycardia*" OR "atrium tachyarrhythmia*" OR "auricular fibrillation" OR "auricular flutter*" OR "auricular tachycardia*" OR "auricular tachyarrhythmia*" OR "Ventricular Premature Beat*" OR VPB* OR "ventricular premature contraction*" OR "Premature Ventricular Complex*" OR "Premature Ventricular Contraction*" OR "Atrial Premature Beat*" OR "Atrial Premature Contraction*" OR "Atrial Premature Complex*" OR "Premature Atrial Contraction*" OR "Abnormal Heart Rhythm*" OR Arrhythmogenesis OR "Atrial Ectopic Beat*" OR Bradycardia OR "Cardiac Rhythm Disturbance*" OR "Heart Rate Variability" OR HRV OR "Heart Block" OR "Irregular Heartbeat*" OR "Long QT Syndrome" OR "Corrected QT Interval" OR "QT Interval" OR "QT Interval Prolongation" OR QTc OR "ECG Abnormalit*" OR "Electrocardiogr* Abnormalit*" OR "ECG Disturbance*" OR "Electrocardiogr* Disturbance*" OR "ECG Alteration*" OR "Electrocardiogr* Alteration*" OR "Electrocardiogr* Change*" OR "ECG Change*" OR "Left Ventricular Dysfunction" OR Proarrhythmi* OR Pro-arrhythmi* OR "Ventricular Electrophysiological Disturbance*" OR "Ventricular Extrasystole*" OR "Heart Rate Abnormali*" OR "Cardiac Autonomic Dysfunction" OR "Cradiac Dysfunction" OR "Heart Dysfunction" OR "Cardiac Abnormalit*" OR "Heart Abnormalit*" OR "Heart Autonomic Dysfunction" OR "Sinus Arrhythmia*" OR "Pre_Excitation Syndrome" OR "Cardiac Reploari*ation" OR "Heart Repolari*ation" OR "Sick Sinus Syndrome" OR "Brugada Syndrome" OR "Premature cardiac Complex" OR "Bundle Branch Block" OR "Atrioventricular block" OR "AV Block" OR "Sinoatrial Block" OR "Cardiac Complex* Premature" OR Parasystole OR "Commotio Cordis") OR TITLE-ABS-KEY(“death” OR "decease" OR “demise” OR “die” OR “dead” OR “loss of life” OR “life time” OR “lifetime” OR “life span” OR “duration of life” OR “mortality” OR “mortalit*” OR “length of life” OR “life duration” OR “longevity” OR “natural life” OR “operating life” OR “survival”))

**Web of knowledge**

[#1](http://www.ncbi.nlm.nih.gov/pubmed/advanced) TS=(hypoglycemia OR hypoglycaemia OR hypogly* OR hyperinsulin* OR hyper-insulin* OR "insulin coma*" OR nesidioblastos* OR "low blood sugar" OR "low blood glucose" OR "Glyc* Variability" OR "Glucose Variability")

[#2](http://www.ncbi.nlm.nih.gov/pubmed/advanced) TS=(NIDDM OR MODY OR “glucose intolerance” OR “late onset diabet*” OR “maturity onset diabet*” OR “non insulin* depend*” OR “noninsulin* depend*” OR “type 2 diabet*” OR “type II diabet*” OR “insulin resistance” OR IDDM OR T1DM OR T1D OR “typ* 1 diabet*” OR “typ* I diabet*” OR “insulin* depend*” OR “insulin?depend*” OR “earl* diabet*” OR “auto?immun* diabet*” OR “sudden onset diabet*” OR “insulin* defic* absolut*” OR “acidos* diabet” OR “juvenil* diabet*” OR “child* diabet*” OR “keto* diabet*” OR “labil* diabet*” OR “britt* diabet*”)

[#3](http://www.ncbi.nlm.nih.gov/pubmed/advanced) TS=("Heart Arrhythmia*" OR "Heart Dysrhythmia*" OR Arrhythmia* OR tachycardia* OR tachyarrhythmia* OR Dysrhythmia* OR "atrial fibrillation" OR "Ventricular fibrillation" OR "atrial flutter*" OR "atrial tachycardia*" OR "supraventricular tachycardia*" OR "supraventricular tachyarrhythmia*" OR "atrial tachyarrhythmia*" OR "Ventricular tachycardia*" OR "Ventricular tachyarrhythmia*" OR AF OR "atrium fibrillation" OR "atrium flutter*" OR "atrium tachycardia*" OR "atrium tachyarrhythmia*" OR "auricular fibrillation" OR "auricular flutter*" OR "auricular tachycardia*" OR "auricular tachyarrhythmia*" OR "Ventricular Premature Beat*" OR VPB* OR "ventricular premature contraction*" OR "Premature Ventricular Complex*" OR "Premature Ventricular Contraction*" OR "Atrial Premature Beat*" OR "Atrial Premature Contraction*" OR "Atrial Premature Complex*" OR "Premature Atrial Contraction*" OR "Abnormal Heart Rhythm*" OR Arrhythmogenesis OR "Atrial Ectopic Beat*" OR Bradycardia OR "Cardiac Rhythm Disturbance*" OR "Heart Rate Variability" OR HRV OR "Heart Block" OR "Irregular Heartbeat*" OR "Long QT Syndrome" OR "Corrected QT Interval" OR "QT Interval" OR "QT Interval Prolongation" OR QTc OR "ECG Abnormalit*" OR "Electrocardiogr* Abnormalit*" OR "ECG Disturbance*" OR "Electrocardiogr* Disturbance*" OR "ECG Alteration*" OR "Electrocardiogr* Alteration*" OR "Electrocardiogr* Change*" OR "ECG Change*" OR "Left Ventricular Dysfunction" OR Proarrhythmi* OR Pro-arrhythmi* OR "Ventricular Electrophysiological Disturbance*" OR "Ventricular Extrasystole*" OR "Heart Rate Abnormali*" OR "Cardiac Autonomic Dysfunction" OR "Cradiac Dysfunction" OR "Heart Dysfunction" OR "Cardiac Abnormalit*" OR "Heart Abnormalit*" OR "Heart Autonomic Dysfunction" OR "Sinus Arrhythmia*" OR "Pre_Excitation Syndrome" OR "Cardiac Reploari*ation" OR "Heart Repolari*ation" OR "Sick Sinus Syndrome" OR "Brugada Syndrome" OR "Premature cardiac Complex" OR "Bundle Branch Block" OR "Atrioventricular block" OR "AV Block" OR "Sinoatrial Block" OR "Cardiac Complex* Premature" OR Parasystole OR "Commotio Cordis" OR “death” OR "decease" OR “demise” OR “die” OR “dead” OR “loss of life” OR “life time” OR “lifetime” OR “life span” OR “duration of life” OR “mortality” OR “mortalit*” OR “length of life” OR “life duration” OR “longevity” OR “natural life” OR “operating life” OR “survival”)

[#4](http://www.ncbi.nlm.nih.gov/pubmed/advanced) [#1](http://www.ncbi.nlm.nih.gov/pubmed/advanced) AND [#2](http://www.ncbi.nlm.nih.gov/pubmed/advanced) AND [#3](http://www.ncbi.nlm.nih.gov/pubmed/advanced)

**CINAHL (EBSCOhost)**

S1 (MH“Hypoglycemia”)

S2 TI(hypoglycemia OR hypoglycaemia OR hypogly* OR hyperinsulin* OR hyper-insulin* OR "insulin coma*" OR nesidioblastos* OR "low blood sugar" OR "low blood glucose" OR "Glyc* Variability" OR "Glucose Variability")

S3 AB(Hypoglycemia OR Hypoglycaemia OR hypogly* OR hyperinsulin* OR hyper-insulin* OR "insulin coma*" OR nesidioblastos* OR "low blood sugar" OR "low blood glucose" OR "Glyc* Variability" OR "Glucose Variability")

S4 OR/S1-S3

S5 (MH“Diabetes mellitus, type 2”)

S6 (MH“glucose intolerance”)

S7 (MH“insulin resistance”)

s8 TI(NIDDM OR MODY OR “glucose intolerance” OR “late onset diabet*” OR “maturity onset diabet*” OR “non insulin* depend*” OR “noninsulin* depend*” OR “type 2 diabet*” OR “type II diabet*” OR “insulin resistance”)

s9 AB(NIDDM OR MODY OR “glucose intolerance” OR “late onset diabet*” OR “maturity onset diabet*” OR “non insulin* depend*” OR “noninsulin* depend*” OR “type 2 diabet*” OR “type II diabet*” OR “insulin resistance”)

S10 OR/S5-S9

S11 (MH"Diabetes mellitus, type 1")

S12 (MH"Diabetic Ketoacidosis")

S13 TI(IDDM OR T1DM OR T1D OR “typ* 1 diabet*” OR “typ* I diabet*” OR “insulin* depend*” OR “insulin?depend*” OR “earl* diabet*” OR “auto?immun* diabet*” OR “sudden onset diabet*” OR “insulin* defic* absolut*” OR “acidos* diabet” OR “juvenil* diabet*” OR “child* diabet*” OR “keto* diabet*” OR “labil* diabet*” OR “britt* diabet*”)

S14 AB(IDDM OR T1DM OR T1D OR “typ* 1 diabet*” OR “typ* I diabet*” OR “insulin* depend*” OR “insulin?depend*” OR “earl* diabet*” OR “auto?immun* diabet*” OR “sudden onset diabet*” OR “insulin* defic* absolut*” OR “acidos* diabet” OR “juvenil* diabet*” OR “child* diabet*” OR “keto* diabet*” OR “labil* diabet*” OR “britt* diabet*”)

S15 OR/S11-S14

S16 S10 OR S15

S17 (MH“[Arrhythmia, Atrial](https://web-p-ebscohost-com-s.webvpn.cams.cn/ehost/mesh/tree?term=Arrhythmia%2C%20Atrial&sid=43d3ef94-48c5-44c3-96e0-4f67ce4e6cef%40redis&vid=8)”)

S18 (MH“Atrial Flutter”)

S19 (MH“Tachycardia, Atrial”)

S20 (MH“Atrial Fibrillation”)

S21 (MH“Ventricular Fibrillation”)

S22 (MH"[Tachycardia, Supraventricular](javascript:XslPostBack('ctl00$ctl00$MainContentArea$MainContentArea$ctrlResults','meshDetail','index%7C1%24term%7CTachycardia%2C%20Supraventricular%24cmd%7CmeshDetail');)")

S23 (MH“[Extrasystole](https://web-s-ebscohost-com-s.webvpn.cams.cn/ehost/mesh/tree?term=Extrasystole&sid=1862e4f4-bafe-48fa-bb0a-5d897599a7ce%40redis&vid=8)”)

S24 (MH“Premature Atrial Contractions”)

S25 (MH“Premature Ventricular Contractions”)

S26 (MH“Ventricular Dysfunction, Left”)

S27 (MH“Bradycardia”)

S28 (MH“Heart Block”)

S29 (MH“Arrhythmia, Sinus”)

S30 (MH“Pre-Excitation Syndromes”)

S31 (MH“Long QT Syndrome”)

S32 (MH“Sick Sinus Syndrome”)

S33 (MH“Brugada Syndrome”)

S34 TI("Heart Arrhythmia*" OR "Heart Dysrhythmia*" OR Arrhythmia* OR tachycardia* OR tachyarrhythmia* OR Dysrhythmia* OR "atrial fibrillation" OR "Ventricular fibrillation" OR "atrial flutter*" OR "atrial tachycardia*" OR "supraventricular tachycardia*" OR "supraventricular tachyarrhythmia*" OR "atrial tachyarrhythmia*" OR "Ventricular tachycardia*" OR "Ventricular tachyarrhythmia*" OR "atrium fibrillation" OR "atrium flutter*" OR "atrium tachycardia*" OR "atrium tachyarrhythmia*" OR "auricular fibrillation" OR "auricular flutter*" OR "auricular tachycardia*" OR "auricular tachyarrhythmia*" OR "Ventricular Premature Beat*" OR VPB* OR "ventricular premature contraction*" OR "Premature Ventricular Complex*" OR "Premature Ventricular Contraction*" OR "Atrial Premature Beat*" OR "Atrial Premature Contraction*" OR "Atrial Premature Complex*" OR "Premature Atrial Contraction*" OR "Abnormal Heart Rhythm*" OR Arrhythmogenesis OR "Atrial Ectopic Beat*" OR Bradycardia OR "Cardiac Rhythm Disturbance*" OR "Heart Rate Variability" OR HRV OR "Heart Block" OR "Irregular Heartbeat*" OR "Long QT Syndrome" OR "Corrected QT Interval" OR "QT Interval" OR "QT Interval Prolongation" OR QTc OR "ECG Abnormalit*" OR "Electrocardiogr* Abnormalit*" OR "ECG Disturbance*" OR "Electrocardiogr* Disturbance*" OR "ECG Alteration*" OR "Electrocardiogr* Alteration*" OR "Electrocardiogr* Change*" OR "ECG Change*" OR "Left Ventricular Dysfunction" OR Proarrhythmi* OR Pro-arrhythmi* OR "Ventricular Electrophysiological Disturbance*" OR "Ventricular Extrasystole*" OR "Heart Rate Abnormali*" OR "Cardiac Autonomic Dysfunction" OR "Cradiac Dysfunction" OR "Heart Dysfunction" OR "Cardiac Abnormalit*" OR "Heart Abnormalit*" OR "Heart Autonomic Dysfunction" OR "Sinus Arrhythmia*" OR "Pre_Excitation Syndrome" OR "Cardiac Reploari*ation" OR "Heart Repolari?ation" OR "Sick Sinus Syndrome" OR "Brugada Syndrome" OR "Premature cardiac Complex" OR "Bundle Branch Block" OR "Atrioventricular block" OR "AV Block" OR "Sinoatrial Block" OR "Cardiac Complex* Premature" OR Parasystole OR "Commotio Cordis" OR AF)

S35 AB("Heart Arrhythmia*" OR "Heart Dysrhythmia*" OR Arrhythmia* OR tachycardia* OR tachyarrhythmia* OR Dysrhythmia* OR "atrial fibrillation" OR "Ventricular fibrillation" OR "atrial flutter*" OR "atrial tachycardia*" OR "supraventricular tachycardia*" OR "supraventricular tachyarrhythmia*" OR "atrial tachyarrhythmia*" OR "Ventricular tachycardia*" OR "Ventricular tachyarrhythmia*" OR "atrium fibrillation" OR "atrium flutter*" OR "atrium tachycardia*" OR "atrium tachyarrhythmia*" OR "auricular fibrillation" OR "auricular flutter*" OR "auricular tachycardia*" OR "auricular tachyarrhythmia*" OR "Ventricular Premature Beat*" OR VPB* OR "ventricular premature contraction*" OR "Premature Ventricular Complex*" OR "Premature Ventricular Contraction*" OR "Atrial Premature Beat*" OR "Atrial Premature Contraction*" OR "Atrial Premature Complex*" OR "Premature Atrial Contraction*" OR "Abnormal Heart Rhythm*" OR Arrhythmogenesis OR "Atrial Ectopic Beat*" OR Bradycardia OR "Cardiac Rhythm Disturbance*" OR "Heart Rate Variability" OR HRV OR "Heart Block" OR "Irregular Heartbeat*" OR "Long QT Syndrome" OR "Corrected QT Interval" OR "QT Interval" OR "QT Interval Prolongation" OR QTc OR "ECG Abnormalit*" OR "Electrocardiogr* Abnormalit*" OR "ECG Disturbance*" OR "Electrocardiogr* Disturbance*" OR "ECG Alteration*" OR "Electrocardiogr* Alteration*" OR "Electrocardiogr* Change*" OR "ECG Change*" OR "Left Ventricular Dysfunction" OR Proarrhythmi* OR Pro-arrhythmi* OR "Ventricular Electrophysiological Disturbance*" OR "Ventricular Extrasystole*" OR "Heart Rate Abnormali*" OR "Cardiac Autonomic Dysfunction" OR "Cradiac Dysfunction" OR "Heart Dysfunction" OR "Cardiac Abnormalit*" OR "Heart Abnormalit*" OR "Heart Autonomic Dysfunction" OR "Sinus Arrhythmia*" OR "Pre_Excitation Syndrome" OR "Cardiac Reploari*ation" OR "Heart Repolari?ation" OR "Sick Sinus Syndrome" OR "Brugada Syndrome" OR "Premature cardiac Complex" OR "Bundle Branch Block" OR "Atrioventricular block" OR "AV Block" OR "Sinoatrial Block" OR "Cardiac Complex* Premature" OR Parasystole OR "Commotio Cordis" OR AF)

S36 OR/S17-S35

S37 (MH"death")

S38 (MH"mortality")

S39 TI(“death” OR "decease" OR “demise” OR “die” OR “dead” OR “loss of life” OR “life time” OR “lifetime” OR “life span” OR “duration of life” OR “mortality” OR “mortalit*” OR “length of life” OR “life duration” OR “longevity” OR “natural life” OR “operating life” OR “survival”)

S40 AB(“death” OR "decease" OR “demise” OR “die” OR “dead” OR “loss of life” OR “life time” OR “lifetime” OR “life span” OR “duration of life” OR “mortality” OR “mortalit*” OR “length of life” OR “life duration” OR “longevity” OR “natural life” OR “operating life” OR “survival”)

S41 OR/S37-S40

S42 S36 OR S41

S43 S4 AND S16 AND S42

Lim S43 to human
