# Supplementary figures and images for: Association of hypoglycaemia with the risks of arrhythmia and mortality in individuals with diabetes - a systematic review and meta-analysis
Source: Front Endocrinol (Lausanne). 2023 Aug 14;14:1222409. doi: 10.3389/fendo.2023.1222409 (PMC10461564; doi:10.3389/fendo.2023.1222409)

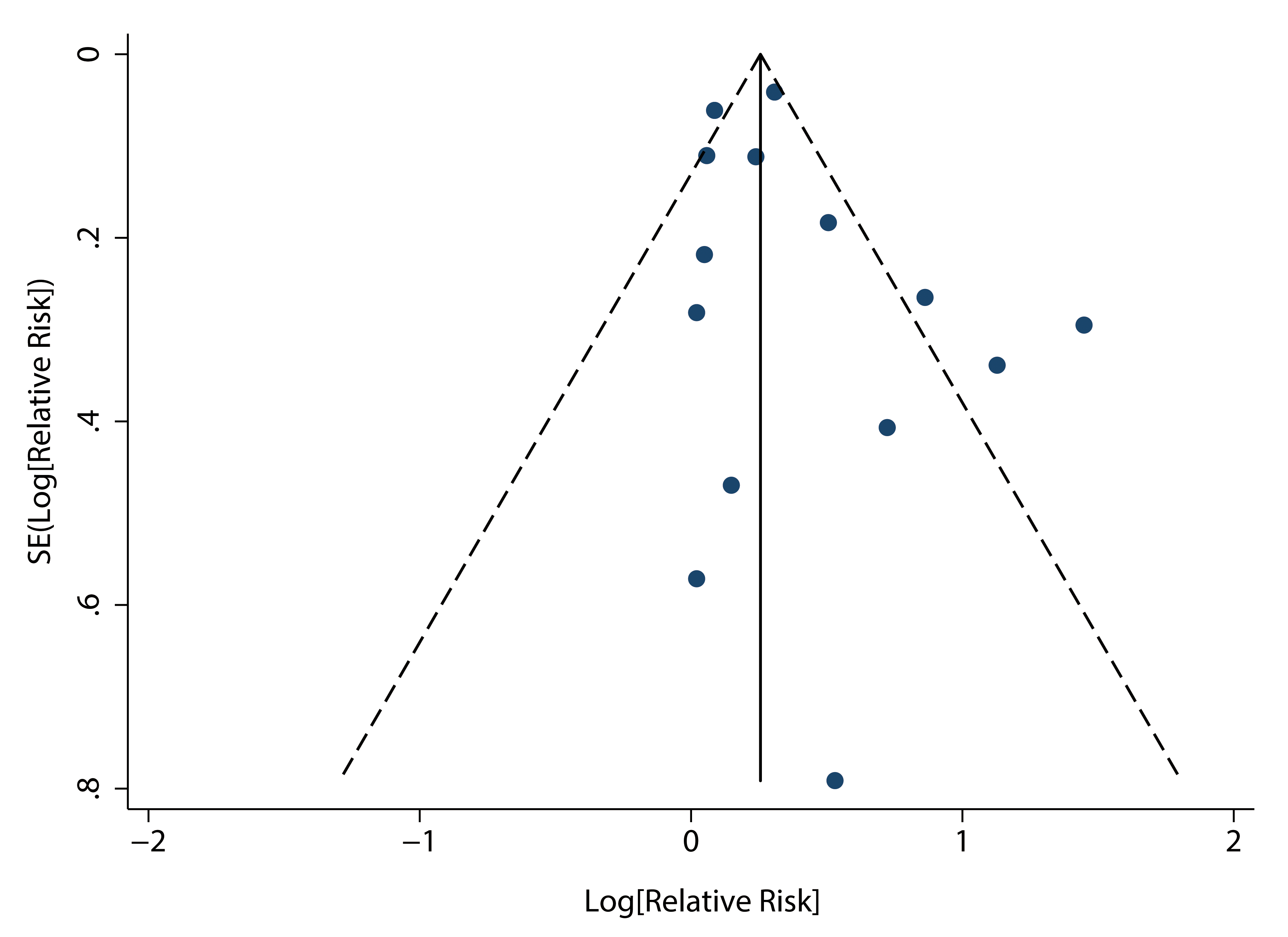

Supplement: Supplementary file 3 [file Image_1.tif]

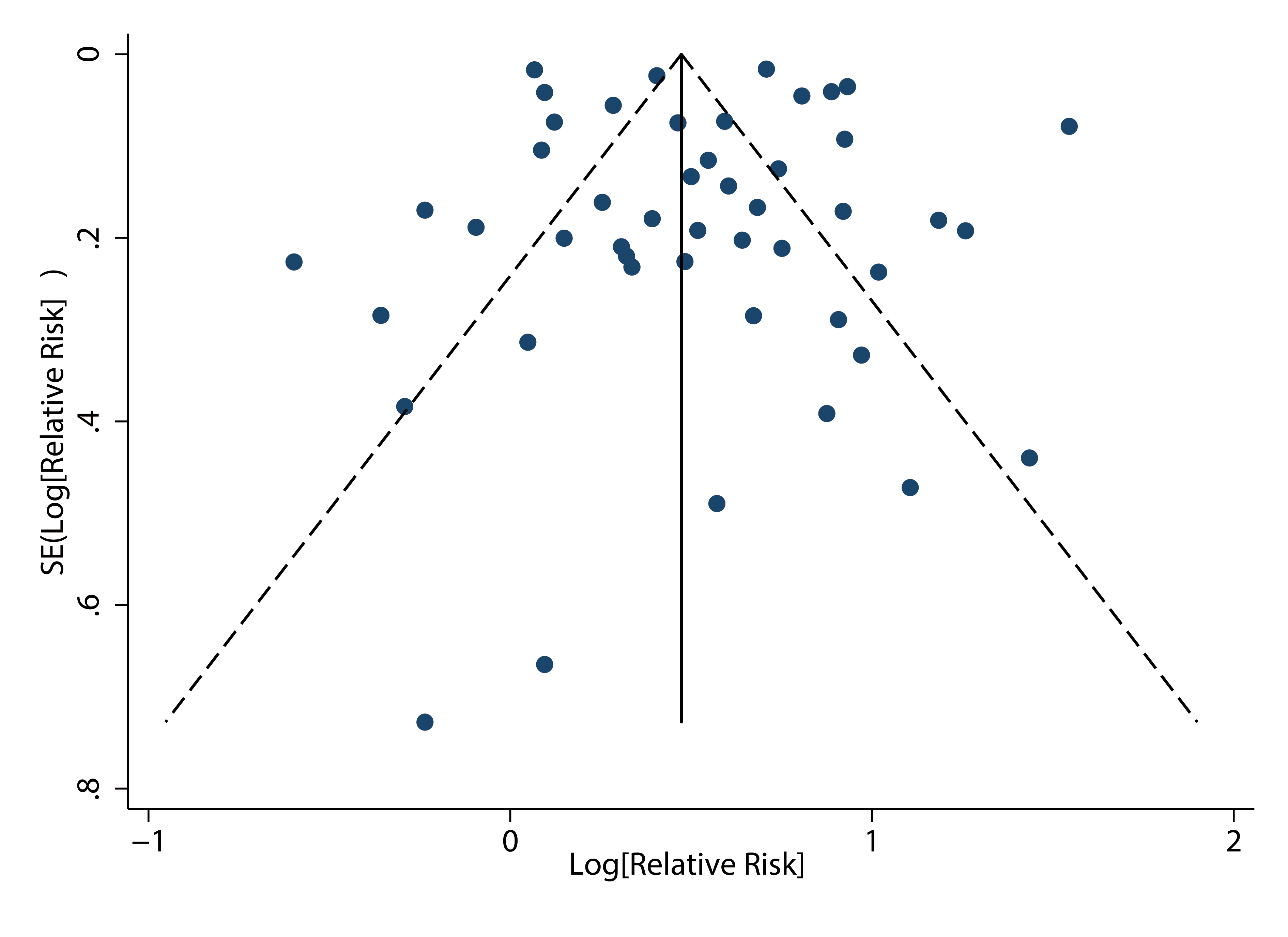

Supplement: Supplementary file 4 [file Image_2.tif]

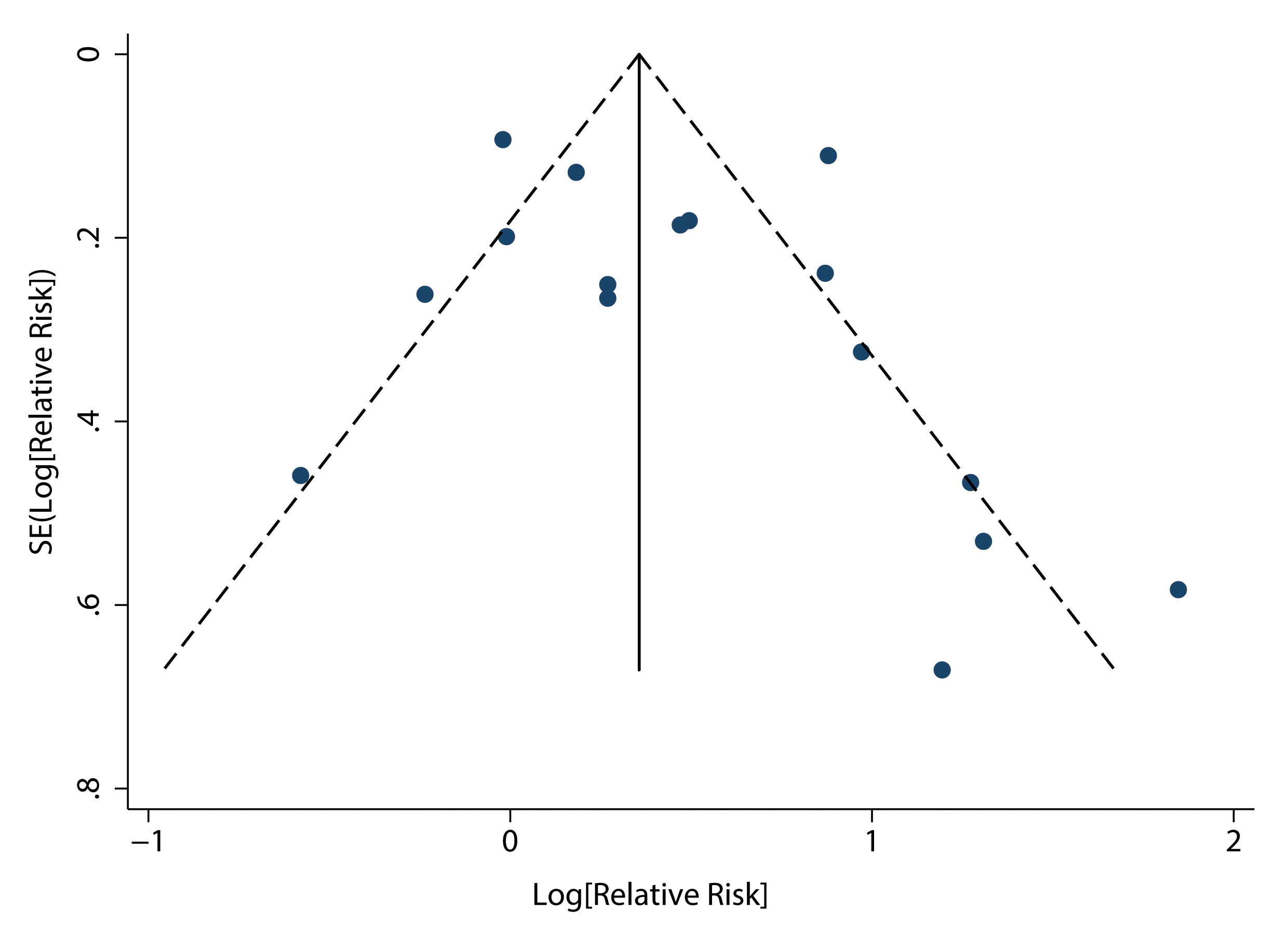

Supplement: Supplementary file 5 [file Image_3.tif]
